# Outcomes of culture-negative vs. culture-positive infective endocarditis: the ESC-EORP EURO-ENDO registry

**DOI:** 10.1093/eurheartj/ehac307

**Published:** 2022-06-08

**Authors:** William K F Kong, Antonio Salsano, Daniele Roberto Giacobbe, Bogdan A Popescu, Cécile Laroche, Xavier Duval, Robert Schueler, Antonella Moreo, Paolo Colonna, Cornelia Piper, Francisco Calvo-Iglesias, Luigi P Badano, Ilija Srdanovic, David Boutoille, Olivier Huttin, Elisabeth Stöhr, Ana Teresa Timóteo, Jolanta Justina Vaskelyte, Anita Sadeghpour, Pilar Tornos, Leila Abid, Kian Keong Poh, Gilbert Habib, Patrizio Lancellotti, the EURO-ENDO Investigators, Gilbert Habib, Patrizio Lancellotti, Bernard Cosyns, Erwan Donal, Paola Erba, Gilbert Habib, Bernard Iung, Aldo P Maggioni, Bogdan A Popescu, Bernard Prendergast, Pilar Tornos, Nora Nabila Ali Tatar-Chentir, Mouaz Al-Mallah, Meriam Astrom Aneq, George Athanassopoulos, Luigi Paolo Badano, Soraya Benyoussef, Erick Calderon Aranda, Nuno Miguel Cardim, Kwan-Leung Chan, Bernard Cosyns, Ines Cruz, Thor Edvardsen, Georg Goliasch, Gilbert Habib, Andreas Hagendorff, Krasimira Hristova, Bernard Iung, Otto Kamp, Duk-Hyun Kang, William Kong, Simon Matskeplishvili, Marwa Meshaal, Maja Mirocevic, Aleksandar N Neskovic, Michal Pazdernik, Edyta Plonska-Gosciniak, Bogdan A Popescu, Bernard Prendergast, Maha Raissouni, Ricardo Ronderos, Leyla Elif Sade, Anita Sadeghpour, Antonia Sambola, Shantanu Sengupta, Jadranka Separovic-Hanzevacki, Masaaki Takeuchi, Edwin Tucay, Ana Clara Tude Rodrigues, Albert Varga, Jolanta Vaskelyte, Kentaro Yamagata, Kyriakos Yiangou, Hosam Zaky, R Ronderos, G Avegliano, P Fernandez Oses, E Filipini, I Granada, A Iribarren, M Mahia, F Nacinovich, S Ressi, R Obregon, M Bangher, J Dho, L Cartasegna, ML Plastino, V Novas, C Shigel, G Reyes, M De Santos, N Gastaldello, M Granillo Fernandez, M Potito, G Streitenberger, P Velazco, JH Casabé, C Cortes, E Guevara, F Salmo, M Seijo, F Weidinger, M Heger, R Brooks, C Stöllberger, C-Y Ho, L Perschy, L Puskas, G Goliasch, C Binder, R Rosenhek, M Schneider, M-P Winter, E Hoffer, M Melissopoulou, E Lecoq, D Legrand, S Jacquet, M Massoz, P Lancellotti, L Pierard, R Dulgheru, S Marchetta, C D´Emal, C Oury, B Cosyns, S Droogmans, D Kerkhove, A Motoc, D Plein, B Roosens, L Soens, C Weytjens, I Lemoine, I Rodrigus, B Paelinck, B Amsel, P Unger, D Konopnicki, C Beauloye, A Pasquet, S Pierard, D Vancraeynest, JL Vanoverschelde, F Sinnaeve, JL Andrade, AC Tude Rodrigues, K Staszko, R Dos Santos Monteiro, MH Miglioranza, DL Shuha, M Alcantara, V Cravo, L Fazzio, A Felix, M Iso, C Musa, AP Siciliano, F Villaca Filho, J Braga, A Rodrigues, R Silva, F Vilela, D Rodrigues, L Silva, S Morhy, C Fischer, R Silva, M Vieira, T Afonso, J Abreu, SN Falcao, V Moises, A Gouvea, G João, F Mancuso, C Silva, AC Souza, CS Abboud, R Bellio de Mattos Barretto, A Ramos, R Arnoni, JE Assef, DJ Della Togna, D Le Bihan, L Miglioli, AP Romero Oliveira, R Tadeu Magro Kroll, D Cortez, CL Gelape, MdC Peirira Nunes, TC De Abreu Ferrari, K-L Chan, K Hay, V Le, M Page, F Poulin, C Sauve, K Serri, C Mercure, J Beaudoin, P Pibarot, I Sebag, L Rudski, G Ricafort, B Barsic, V Krajinovic, M Vargovic, J Separovic-Hanzevacki, D Lovric, V Reskovic-Luksic, J Vincelj, S Jaksic Jurinjak, V Yiannikourides, M Ioannides, C Kyriakou, C Pofaides, V Masoura, K Yiangou, J Pudich, A Linhart, M Siranec, J Marek, K Blechova, M Kamenik, M Pazdernik, R Pelouch, Z Coufal, M Mikulica, M Griva, E Jancova, M Mikulcova, M Taborsky, J Precek, M Jecmenova, J Latal, J Widimsky, T Butta, S Machacek, R Vancata, J Spinar, M Holicka, F Pow Chon Long, N Anzules, A Bajana Carpio, G Largacha, E Penaherrera, D Moreira, E Mahfouz, E Elsafty, A Soliman, Y Zayed, J Aboulenein, M Abdel-Hay, A Almaghraby, M Abdelnaby, M Ahmed, B Hammad, Y Saleh, H Zahran, O Elgebaly, A Saad, M Ali, A Zeid, R El Sharkawy, M Meshaal, A Al Kholy, R Doss, D Osama, H Rizk, A Elmogy, M Mishriky, P Assayag, S El Hatimi, E Botelho-Nevers, S Campisi, J-F Fuzellier, A Gagneux-Brunon, R Pierrard, C Tulane, M Detoc, T Mehalla, D Boutoille, O Al Habash, N Asseray-Madani, C Biron, J Brochard, J Caillon, C Cueff, T Le Tourneau, AS Lecompte, R Lecomte, M Lefebvre, MM Magali Michel, S Pattier, S Delarue, M Le Bras, J Orain, J-F Faucher, V Aboyans, A Beeharry, H Durox, M Lacoste, J Magne, D Mohty, A David, V Pradel, V Sierra, A Neykova, B Bettayeb, S Elkentaoui, B Tzvetkov, G Landry, C Strady, K Ainine, S Baumard, C Brasselet, C Tassigny, V Valente-Pires, M Lefranc, B Hoen, B Lefevre, E Curlier, C Callier, N Fourcade, Y Jobic, S Ansard, R Le Berre, P Le Roux, F Le Ven, M-C Pouliquen, G Prat, F Bouchart, A Savoure, C Alarcon, C Chapuzet, I Gueit, C Tribouilloy, Y Bohbot, F Peugnet, M Gun, B Iung, X Duval, X Lescure, E Ilic-Habensus, N Sadoul, C Selton-Suty, F Alla, E Chevalier, F Goehringer, O Huttin, R Garcia, V Le Marcis, P Tattevin, E Donal, E Flecher, M Revest, G Habib, S Hubert, J-P Casalta, F Gouriet, F Arregle, S Cammilleri, L Tessonnier, A Riberi, C Chirouze, K Bouiller, A-S Brunel, D Fournier, L Hustache-Mathieu, T Klopfenstein, J Moreau, P Lim, L Oliver, J Ternacle, A Moussafeur, P Chavanet, L Piroth, M Buisson, S Mahy, C Martins, A Salmon-Rousseau, S Gohier, C Piper, J Börgermann, D Guckel, D Horstkotte, B Brockmeier, E Winkelmann, A Hagendorff, D Grey, G Nickenig, R Schueler, C Öztürk, E Stöhr, C Hamm, T Walther, R Brandt, A-C Frühauf, CT Hartung, C Hellner, C Wild, M Becker, S Hamada, W Kaestner, K Stangl, F Knebel, G Baldenhofer, A Brecht, H Dreger, C Isner, F Pfafflin, M Stegemann, R Zahn, B Fraiture, C Kilkowski, A-K Karcher, S Klinger, H Tolksdorf, D Tousoulis, C Aggeli, G Sarri, S Sideris, E Venieri, G Athanassopoulos, D Tsiapras, I Armenis, A Koutsiari, G Floros, C Grassos, S Dragasis, L Rallidis, C Varlamos, L Michalis, K Naka, A Bechlioulis, A Kotsia, L Lakkas, K Pappas, C Papadopoulos, S Kiokas, A Lioni, S Misailidou, J Barbetseas, M Bonou, C Kapelios, I Tomprou, K Zerva, A Manolis, E Hamodraka, D Athanasiou, G Haralambidis, L Poulimenos, H Samaras, A Nagy, A Bartykowszki, E Gara, S Sengupta, K Mungulmare, R Kasliwal, M Bansal, A Bhan, S Ranjan, M Kyavar, M Maleki, F Noohi Bezanjani, A Sadeghpour, A Alizadehasl, S Boudagh, A Ghavidel, P Moradnejad, HR Pasha, B Ghadrdoost, D Gilon, J Strahilevitz, S Israel, M Wanounou, C d'Agostino, P Colonna, L De Michele, F Fumarola, M Stante, N Marchionni, V Scheggi, B Alterini, S Del Pace, P Stefano, C Sparano, LP Badano, D Muraru, N Ruozi, R Tenaglia, U Limbruno, A Cresti, P Baratta, M Solari, C Giannattasio, A Moreo, B De Chiara, B Lopez Montero, F Musca, CA Orcese, F Panzeri, CF Russo, F Spano, O Alfieri, M De Bonis, E Agricola, E Busnardo, S Carletti, B Castiglioni, S Chiappetta, B Del Forno, D Ferrara, M Guffanti, G Iaci, E Lapenna, T Nisi, C Oltolini, U Pajoro, R Pasciuta, M Ripa, P Scarpellini, C Tassan Din, R Meneghin, D Schiavi, F Piscione, R Citro, RM Benvenga, L Greco, C Prota, I Radano, L Soriente, M Bellino, D Di Vece, F Santini, A Salsano, GM Olivieri, F Turrini, R Messora, S Tondi, A Olaru, V Agnoletto, L Grassi, C Leonardi, S Sansoni, S Del Ponte, GM Actis Dato, A De Martino, N Ohte, S Kikuchi, K Wakami, K Aonuma, Y Seo, T Ishizu, T Machino-Ohtsuka, M Yamamoto, N Iida, H Nakajima, Y Nakagawa, C Izumi, M Amano, M Miyake, K Takahashi, I Shiojima, Y Miyasaka, H Maeba, Y Suwa, N Taniguchi, S Tsujimoto, T Kitai, M Ota, S Yuda, S Sasaki, N Hagiwara, K Yamazaki, K Ashihara, K Arai, C Saitou, S Saitou, G Suzuki, Y Shibata, N Watanabe, S Nishino, K Ashikaga, N Kuriyama, K Mahara, K Abe, H Fujimaki, T Okubo, H Shitan, S Takanashi, M Terada, H Yamamoto, M Sata, H Yamada, K Kusunose, Y Saijo, H Seno, O Yuichiro, Y Sakata, H Mizuno, S Nakatani, T Onishi, K Sengoku, F Sera, SW Park, K Eun Kyoung, L Ga Yeon, J-w Hwang, C Jin-Oh, S-J Park, L Sang-Chol, C Sung-A, SY Jang, D-H Kang, R Heo, S Lee, J-M Song, E Jung, J Plisiene, A Dambrauskaite, G Gruodyte, R Jonkaitiene, J Vaskelyte, V Mizariene, J Atkocaityte, R Zvirblyte, R Sow, A Codreanu, ECL De la Vega, C Michaux, T Staub, L Jacobs-Orazi, C Mallia Azzopardi, RG Xuereb, T Piscopo, D Borg, R Casha, J Farrugia, M Fenech, E Pllaha, C Vella, K Yamagata, L Grib, E Raevschi, A Grejdieru, G Balan, I Cardaniuc, L Cardaniuc, V Corcea, A Feodorovici, V Gaina, L Girbu, P Jimbei, D Kravcenco, E Panfile, E Prisacari, E Samohvalov, S Samohvalov, N Sceglova, I Benesco, V Marian, N Sumarga, M Mirocevic, B Bozovic, N Bulatovic, P Lakovic, L Music, J Roos-Hesselink, R Budde, T Gamela, A Wahadat, O Kamp, T Meijers, JP Van Melle, VM Deursen, H Crijns, S Bekkers, E Cheriex, M Gilbers, B Kietselaer, C Knackstedt, R Lorusso, S Schalla, S Streukens, S Chamuleau, M-J Cramer, A Teske, T Van der Spoel, A Wind, O Liesbek, J Lokhorst, H Van Heusden, W Tanis, I Van der Bilt, J Vriend, H De Lange-van Bruggen, E Karijodikoro, R Riezebos, E van Dongen, J Schoep, V Stolk, O Axler, F Baumann, S Lebras, T Edvardsen, JT Offstad, JO Beitnes, T Helle-Valle, H Skulstad, R Skardal, N Qamar, S Furnaz, B Ahmed, MH Butt, MF Khanzada, T Saghir, A Wahid, T Hryniewiecki, P Szymanski, K Marzec, M Misztal-Ogonowska, W Kosmala, M Przewlocka-Kosmala, A Rojek, K Woznicka, J Zachwyc, A Lisowska, M Kaminska, J Kasprzak, E Kowalczyk, DF Strzecka, P Wejner-Mik, M Trabulo, P Freitas, S Ranchordas, G Rodrigues, P Pinto, C Queiros, J Azevedo, L Marques, D Seabra, L Branco, J Abreu, M Cruz, A Galrinho, R Moreira, P Rio, AT Timoteo, M Selas, NM Cardim, V Carmelo, B Duque Neves, H Pereira, I Cruz, A Guerra, A Marques, I Pintassilgo, MC Tomescu, N-M Trofenciuc, M Andor, A Bordejevic, HS Branea, F Caruntu, L Cirin, IM Citu, CA Cotoraci, D Darabantiu, R Farcas, I Marincu, A Mavrea, MF Onel, T Parvanescu, D Pop, AL Pop-Moldovan, MI Puticiu, LA Velcean, A Ionac, D Cozma, C Mornos, F Goanta, I Popescu, R Beyer, R Mada, R Rancea, H Rosianu, R Tomoaia, C Stanescu, Z Kobalava, J Karaulova, E Kotova, A Milto, A Pisaryuk, N Povalyaev, M Sorokina, J Alrahimi, A Elshiekh, A Jamiel, A Ahmed, M Al-Mallah, N Attia, B Putnikovic, A Neskovic, A Dimic, B Ivanovic, S Matic, D Trifunovic, J Petrovic, D Kosevic, P Dabic, P Milojevic, I Petrovic, I Stojanovic, I Srdanovic, M Kovacevic, A Redzek, M Stefanovic, S Susak, L Velicki, A Vulin, TC Yeo, W KF Kong, KK Poh, I Vilacosta, M Abd El- Nasser, C Ferrera, C Olmos, F Calvo Iglesias, E Blanco-Gonzalez, M Bravo Amaro, AN Germinas, E Lopez-Rodriguez, J Lugo Adan, P Pazos-Lopez, M Pereira Loureiro, MT Perez, S Raposeiras-Roubin, S Rasheed Yas, M-M Suarez-Varela, F Vasallo Vidal, D Garcia-Dorado, A Sambola, N Fernandez-Hidalgo, T Gonzalez-Alujas, J Lozano, O Maisterra, N Pizzi, R Rios, P Tornos, A Bayes-Genis, L Pedro Botet, N Vallejo, E Berastegui, C Llibre, L Mateu, R Nunez, D Quesada, D Bosch Portell, J Aboal Vinas, X Albert Bertran, R Brugada Tarradellas, P Loma-Osorio Ricon, C Tiron de Llano, MA Arnau, A Bel, M Blanes, A Osa, M Anguita, F Carrasco, J Castillo, JL Zamorano, JL Moya Mur, M Alvaro, C Fernandez-Golfin, JM Monteagudo, E Navas Elorza, MC Farinas Alvarez, J Aguero Balbin, C Arminanzas, F Arnaiz de las Revillas, A Arnaiz Garcia, M Cobo Belaustegui, M Fernandez Sampedro, M Gutierrez Cuadra, JF Gutierrez-Diez, J Zarauza, L Garcia Cuello, C Gonzalez Rico, R Rodriguez-Alvarez, J Goikoetxea, M Montejo, J Miro, M Almela, J Ambrosioni, C Falces, D Fuster, C Garcia-de-la-Maria, M Hernandez-Meneses, J Llopis, F Marco, A Moreno, E Quintana, E Sandoval, A Tellez, JM Tolosana, B Vidal, I Ruiz-Zamora, A Bardaji Ruiz, E Sanz Girgas, G Garcia-Pardo, M Guillen Marzo, A Rodriguez Oviedo, A Villares Jimenez, L Abid, R Hammami, S Kammoun, MS Mourali, F Mghaieth Zghal, M Ben Hlima, S Boudiche, S Ouali, L Zakhama, S Antit, I Slama, O Gulel, M Sahin, LE Sade, E Karacaglar, S Kucukoglu, O Cetinarslan, US Yasar, U Canpolat, B Mutlu, H Atas, R Dervishova, C Ileri, H Zaky, J Alhashmi, F Baslib, J Tahir, P Zarger, S Woldman, L Menezes, C Primus, R Uppal, I Bvekerwa, B Chandrasekaran, A Kopanska, B Prendergast, S Cannata, J Chambers, J Hancock, J Klein, R Rajani, MP Ursi, R Dworakowski, A Fife, J Breeze, M Browne-Morgan, M Gunning, S Streather, F Asch, M Zemedkun, B Alyavi, J Uzokov

**Affiliations:** Department of Cardiology, National University Heart Centre Singapore, Singapore; Yong Loo Lin School of Medicine, National University of Singapore, Singapore; Department of Integrated Surgical and Diagnostic Sciences (DISC), University of Genoa, Genoa, Italy; Division of Cardiac Surgery, Ospedale Policlinico San Martino—IRCCS, Largo Rosanna Benzi, 10, Genoa, Italy; Department of Health Sciences (DISSAL), University of Genoa, Genoa, Italy; Infectious Diseases Unit, Ospedale Policlinico San Martino—IRCCS, Genoa, Italy; Department of Cardiology, University of Medicine and Pharmacy ‘Carol Davila’ Euroecolab, Emergency Institute for Cardiovascular Diseases ‘Prof. Dr. C. C. Iliescu’, Bucharest, Romania; EORP, European Society of Cardiology, Sophia-Antipolis, France; INSERM Clinical Investigation Center 1425, Université Paris Diderot, Sorbonne Paris-Cité, IAME 1138, Paris, France; AEPEI Service de Maladies Infectieuses et Tropicales, APHP, Hôpital Bichat, Paris, France; Heart Center, University Hospital, Bonn, Germany; Dipartimento CardioToracoVascolare ‘De Gasperis’, ASST Grande Ospedale Metropolitano Niguarda, Milan, Italy; Cardiology Hospital, Policlinico University Hospital of Bari, Bari, Italy; Clinic for General and Interventional Cardiology/Angiology, Herz-und Diabeteszentrum Nordrhein-Westfalen, Ruhr-Universität Bochum, Bad Oeynhausen, Germany; Departamento de Cardiología, Hospital Álvaro Cunqueiro, Vigo, Pontevedra, Spain; University of Milano-Bicocca, Milano, Italy; Department of Cardiovascular, Neural and Metabolic Sciences; Istituto Auxologico Italiano, IRCCS—San Luca Hospital, Milano, Italy; Faculty of Medicine, University of Novi Sad, Novi Sad, Serbia; Institute of Cardiovascular Diseases Vojvodina, Sremska Kamenica, Serbia; Department of Infectious Diseases, CIC UIC 1413 INSERM, University Hospital, Nantes, France; Service de Cardiologie, Institut Lorrain du Cœur et des Vaisseaux, CHU de Nancy, Nancy, France; CIC-Plurithématique 1433, Inserm U1116, CHRU Nancy, Université de Lorraine, CIC-IT, U1433, CHRU de Nancy, France; INSERM U1254, IADI, Université de Lorraine, Nancy, France; Heart Center, University Hospital, Bonn, Germany; Secretária-Geral Sociedade Portuguesa Cardiologia, Lisbon, Portugal; Assistente Hospitalar Graduada Cardiologia, Hospital Santa Marta, Centro Hospitalar Universitário Lisbon Central, Lisbon, Portugal; NOVA Medical School, Lisbon, Portugal; Department of Cardiology, Lithuanian University of Health Sciences, Kaunas, Lithuania; Rajaie Cardiovascular Medical & Research Center, Tehran, Iran; Duke Cardiovascular MR Center, Durham, NC, USA; Cardiology Service, Hospital Quiron, Barcelona, Spain; Hédi Chaker Hospital, Sfax, Tunisia; Department of Cardiology, National University Heart Centre Singapore, Singapore; Yong Loo Lin School of Medicine, National University of Singapore, Singapore; Department of Cardiology, APHM, La Timone Hospital, Marseille, France; University Hospital of Liege (CHU), Liege, Belgium; Rajaie Cardiovascular Medical & Research Center, Tehran, Iran

**Keywords:** Endocarditis, Infective endocarditis, Heart valves, Blood culture-negative endocarditis, Diagnosis, Surgery

## Abstract

**Aim:**

Fatality of infective endocarditis (IE) is high worldwide, and its diagnosis remains a challenge. The objective of the present study was to compare the clinical characteristics and outcomes of patients with culture-positive (CPIE) vs. culture-negative IE (CNIE).

**Methods and results:**

This was an ancillary analysis of the ESC-EORP EURO-ENDO registry. Overall, 3113 patients who were diagnosed with IE during the study period were included in the present study. Of these, 2590 (83.2%) had CPIE, whereas 523 (16.8%) had CNIE. As many as 1488 (48.1%) patients underwent cardiac surgery during the index hospitalization, 1259 (48.8%) with CPIE and 229 (44.5%) with CNIE. The CNIE was a predictor of 1-year mortality [hazard ratio (HR) 1.28, 95% confidence interval (CI) 1.04–1.56], whereas surgery was significantly associated with survival (HR 0.49, 95% CI 0.41–0.58). The 1-year mortality was significantly higher in CNIE than CPIE patients in the medical subgroup, but it was not significantly different in CNIE vs. CPIE patients who underwent surgery.

**Conclusion:**

The present analysis of the EURO-ENDO registry confirms a higher long-term mortality in patients with CNIE compared with patients with CPIE. This difference was present in patients receiving medical therapy alone and not in those who underwent surgery, with surgery being associated with reduced mortality. Additional efforts are required both to improve the aetiological diagnosis of IE and identify CNIE cases early before progressive disease potentially contraindicates surgery.

## Teaser

CPIE, culture-positive infective endocarditis; CNIE, culture-negative infective endocarditis; ESC-EORP EURO-ENDO, European Infective Endocarditis.

## Structured Graphical Abstract

**Structured Graphical Abstract:**
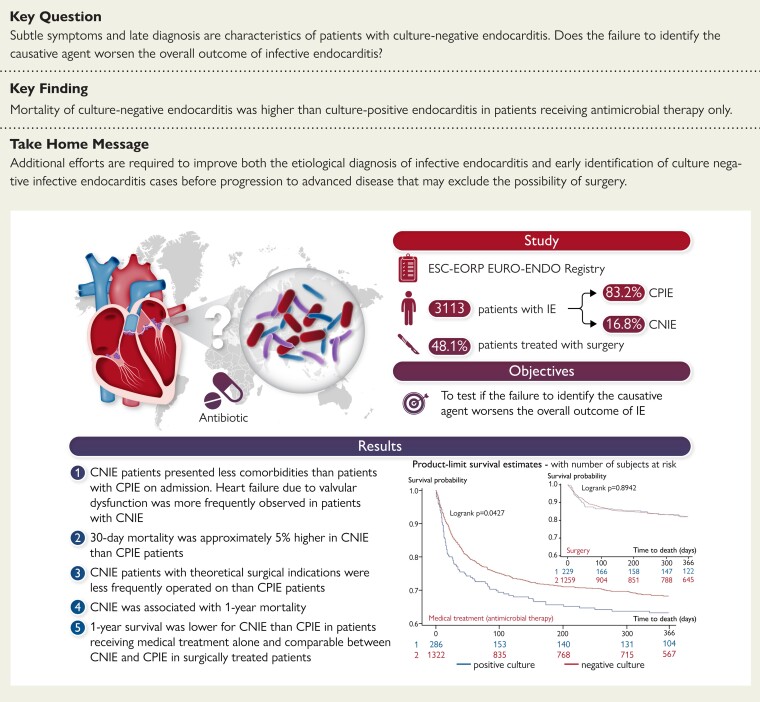
CPIE, culture-positive infective endocarditis; CNIE, culture-negative infective endocarditis; ESC-EORP EURO-ENDO, European Infective Endocarditis.

**See the editorial comment for this article ‘A double negative: culture-negative infective endocarditis’, by E.L. Fosbøl, https://doi.org/10.1093/eurheartj/ehac230.**

## Introduction

Fatality of infective endocarditis (IE) is high worldwide and its diagnosis remains a challenge.^1^ The microbiological profile of IE varies from country to country and across different centres in the same country.^1^ This heterogeneity may reflect the local epidemiology of IE, the diagnostic criteria used, the practice of initiation of antibiotics prior to collecting blood cultures, and the protocols used to pursue aetiological diagnosis.

Identification of the causative microorganism is crucial to select an appropriate targeted antibiotic therapy that, together with surgical debridement whenever indicated, represents the mainstay of the therapeutic approach to IE. With these premises, we hypothesized that: (i) failure to identify the causative agent may be one of the factors which worsens the overall outcome of IE; (ii) recognition of particular characteristics of IE patients without an aetiological diagnosis could help to identify modifiable factors to improve either diagnostic yield or patient health.

Guided by the hypotheses that the negativity of blood cultures may impair the chances to treat these patients adequately (both by delaying and by forcing empirical rather than targeted antibiotic therapy), we conducted an ancillary multicentre study within the European Society of Cardiology (ESC)-EURObservational Research Programme (EORP) European Endocarditis (EURO-ENDO) international registry to compare the clinical characteristics, 30-day mortality, and 1-year outcome of patients with culture-positive (CPIE) vs. culture-negative IE (CNIE).

## Methods

The ESC-EORP EURO-ENDO records the data from the largest cohort of patients admitted to hospitals in Europe and ESC-affiliated/non-affiliated countries and diagnosed with definite or possible IE. The detailed design, study methodology, and definitions of EURO-ENDO have been reported previously.^2^ Briefly, from 1 January 2016 to 31 March 2019, all patients aged 18 years or older with active IE from sites participating in the registry were included in the present study. All centres were asked to include patients during a 1-year period, with a maximum follow-up of 1 year after discharge for each patient. Each centre could participate for a maximum of 2 years. A total of 156 centres from 40 countries included 3113 cases of IE, with a mean of 20.19 patients per centre per year. Among the 156 active centres, 120 (76.9%) were from ESC-affiliated countries and 36 (23.1%) were outside Europe. There were 79.5% high-volume centres and 20.5% low-volume centres.^2^

The primary endpoints were 30-day and 1-year mortality.

### Definitions

The CPIE was defined as IE with the causative agent identified from blood cultures and/or tissue cultures from the excised valve or vegetation, whereas CNIE was defined as IE with negative blood cultures and negative tissue cultures. For the purpose of the study (comparing patients with and without an aetiological diagnosis), patients with positive immunoglobulin G antibodies for *Coxiella burnetii* were included in the CPIE group, although they had negative cultures.

### Data collection

Data were recorded at patient admission, during hospitalization and at follow-up. The following information was collected: demographic and clinical data [age, sex, weight, height, Charlson comorbidity index, date and timing of first signs and symptoms related to the infectious process, underlying cardiac disease, at-risk situation or procedure, Roth’s spots, temperature, Janeway’s lesions, conjunctival haemorrhages, cardiac murmur, heart failure signs, neurological complication, septic shock, atrioventricular (AV) block]; laboratory and microbiological data (creatinine, haemoglobin, white cell count, platelet count; culture results); echocardiographic data (vegetation, abscess, pseudoaneurysm, valvular and perivalvular lesions, valve regurgitation or stenosis); imaging techniques performed at admission and during hospitalization [computed tomography (CT) scan, positron emission tomography (PET)/CT, and cerebral or cardiac magnetic resonance imaging (MRI)]; treatment (antibiotic therapy before admission and during hospitalization, non-antibiotic treatments).^1,3^

The following was also registered: mortality and cause of death at discharge; 30-day mortality; embolic events, infectious and haemodynamic complications at 30-day follow-up; New York Heart Association Classes III and IV at follow-up; recurrence of IE at follow-up; reoperation at follow-up; 1-year mortality.^3,4^

### Data management and statistical analysis

National coordinators, in conjunction with local centres, managed the approvals of national or regional ethics committees or Institutional Review Boards, according to local regulations.

Data were collected by all investigators at the participating centres and included in the format of an electronic case report form for online data entry. All patients enrolled with definite or possible IE were included in the analyses. Continuous variables are expressed as mean (standard deviation) and compared using the Kruskal–Wallis test. Categorical variables are presented as number (percentage) and compared using Pearson’s χ^2^ test or Fisher’s exact test (†) if any expected cell count was <5. Kaplan–Meier curves for all-cause mortality were created and the curves compared using the log-rank test. Univariable analyses of 30-day and 1-year mortality were performed using a Cox proportional hazard model. Variables with *P* < 0.05 were entered in multivariable adjusted Cox proportional hazard models with a backward selection procedure and significance level of *P* = 0.05. In order to test our hypothesis that higher mortality rates in patients with CNIE mainly depend on the risk profile and patient characteristics, the variable ‘culture negative vs. culture positive’ was forced into the final multivariable statistical models. Goodness of fit proposed by May and Hosmer and concordance were calculated to verify the adequacy of the models. In addition, proportional hazard ratio (HR) assumptions were verified graphically and with the Schoenfeld residuals test.

Sensitivity analyses were performed: (i) on 30-day and 1-year mortality in medical and surgical subgroups using multivariable Cox proportional hazard model based on least absolute shrinkage and selection operator (LASSO) penalty for variable selection, starting from the clinically relevant variables detected through univariable analyses; (ii) on the comparison between CNIE and CPIE due to *Staphylococcus aureus*, traditionally associated with poor outcome.^5^

An alpha level <0.05 was considered significant. All analyses were performed using SAS statistical software version 9.4 (SAS Institute, Inc., Cary, NC, USA) and glmnet 4.1-2 package of R software (version 3.6.2; R Foundation for Statistical Computing, Vienna, Austria).

## Results

Overall, 3113 patients who were diagnosed with IE during the study period were included in the present analysis. Of these, 2590 (83.2%) had CPIE, whereas 523 (16.8%) had CNIE. Among patients with CPIE, only 80/2590 (3.1%) were classified as CPIE based only on valve culture results (i.e. they had negative blood cultures). According to the 2015 ESC diagnostic criteria, 92.7% (2401/2590) of CPIE patients had definite IE and 7.3% (189/2590) possible IE, whereas 39.4% (206/523) of CNIE patients had definite IE and 60.6% (317/523) possible IE (*P* < 0.001, see Supplementary material online, *Table S1*). There were 381/2470 (15.4%) CNIE in IE patients from countries affiliated to the ESC and 142/643 (22.1%) CNIE in patients with IE from other countries (*P* < 0.01). Overall, 1488/3096 (48.1%) patients underwent cardiac surgery during the index hospitalization, 1259/2581 (48.8%) with CPIE and 229/515 with CNIE (44.5%). Of note, 22 patients classified as CNIE had positive polymerase chain reaction (PCR) for aetiological agents on the excised valve.

*Table 1* outlines the baseline characteristics (overall and stratified) of patients with CPIE and CNIE. Those with CPIE were older and presented more frequently with ischaemic heart disease (22.3 vs. 17.5%, *P* = 0.02), diabetes mellitus (23.5 vs. 18.4%, *P* = 0.01), and hypertension (49.4 vs. 42.6%, *P* = 0.005). Imaging techniques such as fluorodeoxyglucose (FDG)-PET/CT scan, MRI, and multi-slice CT were more frequently performed in patients with CPIE than in patients with CNIE. The most common site of IE was the aortic valve (49.5%) followed by the mitral valve (42.0%). There were no significant differences in the prevalence of aortic, mitral, pulmonary, tricuspid, and device-related endocarditis between CPIE and CNIE patients (see Supplementary material online, *Table S2*).

**Table 1:** Baseline clinical characteristics of patients with culture-positive vs. culture-negative infective endocarditis

Variable	Total *N* = 3113	CPIE *N* = 2590 (83.2%)	CNIE *N* = 523 (16.8%)	*P*-value
Male sex	2144/3113 (68.9%)	1808/2590 (69.8%)	336/523 (64.2%)	0.01
Age (years)	59.3 (±18.0)	60.2 (±17.6)	54.3 (±19.2)	<0.001
Age ≥65 years	1443/3113 (46.4%)	1254/2590 (48.4%)	189/523 (36.1%)	<0.001
**Body mass index (kg/m²)**				
(*N* = 2736)	25.8 (±6.4)	25.9 (±6.5)	25.4 (±5.8)	0.12
Heart failure	661/2837 (23.3%)	536/2337 (22.9%)	125/500 (25.0%)	0.32
Ischaemic heart disease (CAD)	620/2894 (21.4%)	532/2390 (22.3%)	88/504 (17.5%)	0.02
Pre-existing valvular disease	1067/3113 (34.3%)	894/2590 (34.5%)	173/523 (33.1%)	0.53
Cancer	359/3085 (11.6%)	310/2571 (12.1%)	49/514 (9.5%)	0.10
Previous stroke/transient ischaemic attack	340/2857 (11.9%)	280/2351 (11.9%)	60/506 (11.9%)	0.97
Diabetes mellitus	704/3109 (22.6%)	608/2586 (23.5%)	96/523 (18.4%)	0.01
Arterial hypertension	1499/3108 (48.2%)	1276/2585 (49.4%)	223/523 (42.6%)	0.005
Congenital disease	365/3111 (11.7%)	283/2588 (10.9%)	82/523 (15.7%)	0.002
Previous valvular intervention (prosthesis/repair/TAVI)	1023/3113 (32.9%)	865/2590 (33.4%)	158/523 (30.2%)	0.16
Previous procedure—TAVI	70/474 (14.8%)	66/427 (15.5%)	4/47 (8.5%)	0.20
Previous procedure—TMVR	2/95 (2.1%)	2/81 (2.5%)	0/14 (0.0%)	1.0
Previous endocarditis	274/3113 (8.8%)	232/2590 (9.0%)	42/523 (8.0%)	0.49
COPD/asthma	317/3108 (10.2%)	264/2586 (10.2%)	53/522 (10.2%)	0.97
Chronic renal failure	551/3110 (17.7%)	468/2587 (18.1%)	83/523 (15.9%)	0.22
LVEF (*N* = 2657)	55.6 (±12.0)	55.9 (±11.8)	54.5 (±12.7)	0.02
Echocardiography	2827/2832 (99.8%)	2329/2333 (99.8%)	498/499 (99.8%)	1.0
FDG-PET/CT scan	518/3113 (16.6%)	463/2590 (17.9%)	55/523 (10.5%)	<0.001
Magnetic resonance imaging	581/3113 (18.7%)	520/2590 (20.1%)	61/523 (11.7%)	<0.001
Leucocyte scintigraphy	38/3113 (1.2%)	32/2590 (1.2%)	6/523 (1.1%)	0.87
Multi-slice computed tomography	1656/3113 (53.2%)	1401/2590 (54.1%)	255/523 (48.8%)	0.03

CAD, coronary artery disease; COPD, chronic obstructive pulmonary disease; CNIE, culture-negative infective endocarditis; CPIE, culture-positive infective endocarditis; FDG-PET/CT, fluorodeoxyglucose positron emission tomography/computed tomography; LVEF, left ventricular ejection fraction; TAVI, transcatheter aortic valve implantation; TMVR, transcatheter mitral valve repair.

Signs and symptoms of IE in patients with CPIE vs. CNIE are presented in Supplementary material online, *Table S2*. On admission, fever (31.5 vs. 26.8%, *P* = 0.04), septic shock (7.0 vs. 4.4%, *P* = 0.03), and spondylitis (6.1 vs. 1.7%, *P* < 0.001) were more frequently observed in patients with CPIE than in those with CNIE. Conversely, CNIE patients presented more frequently with cardiac murmur (63.3 vs. 70.2%, *P* = 0.003) and congestive heart failure (26.4 vs. 31.0%, *P* = 0.03).

All patients were treated with antimicrobial therapies. Details about medical treatment are shown in Supplementary material online, *Table S3*. Before the index admission for IE, patients with CNIE received a slightly higher number of antimicrobials than CPIE patients (mean 0.9 vs. 0.7, *P* = 0.01), whereas CPIE patients received a higher number of antimicrobials than patients with CNIE (mean 3.5 vs. 3.1, *P* < 0.01) during the index hospitalization. The most frequent antimicrobials administered before the index admission were ampicillin/amoxicillin (12.3%) and quinolones (9.9%). Stratifying by culture results, patients with CNIE were more frequently treated with ceftriaxone (12.1 vs. 6.4%, *P* < 0.001) and vancomycin (12.6 vs. 6.6%, *P* < 0.001) than patients with CPIE. During the index admission for IE, various differences in the types of administered antimicrobials were observed between CPIE and CNIE patients, likely reflecting microorganism-oriented targeted therapy in the CPIE group. Specifically, penicillin, ceftriaxone, oxacillin, rifampicin, clindamycin, cotrimoxazole, and cefazolin were administered more frequently to CPIE than CNIE patients, whereas vancomycin, doxycycline, and carbapenems were more frequently administered to CNIE patients. Microorganisms identified in CPIE were detailed in a previous publication.^2^

At discharge, adverse event such as heart failure (17.9 vs. 13.7%, *P* = 0.02) and valvular dysfunction (20.8 vs. 16.3%, *P* = 0.02) was more frequently observed in CNIE than CPIE patients. Mean length of in-hospital stay was 42.1 days and in-hospital mortality was higher in patients with CNIE than in patients with CPIE (20.1 vs. 16.4%, *P* = 0.04; *Table 2*).

**Table 2:** Adverse events at discharge and short- and long-term mortality results in patients with culture-positive vs. culture-negative endocarditis

Variable	Total *N* = 3113	CPIE *N* = 2590 (83.2%)	CNIE *N* = 523 (16.8%)	*P*-value
Embolic events	641/3113 (20.6%)	561/2590 (21.7%)	80/523 (15.3%)	0.001
Spondylitis	145/3113 (4.7%)	138/2590 (5.3%)	7/523 (1.3%)	<0.001
Cardiogenic shock	189/2837 (6.7%)	149/2337 (6.4%)	40/500 (8.0%)	0.19
Septic shock	287/3113 (9.2%)	246/2590 (9.5%)	41/523 (7.8%)	0.23
Glomerulonephritis	89/3094 (2.9%)	69/2571 (2.7%)	20/523 (3.8%)	0.16
Cerebral haemorrhage	71/3113 (2.3%)	61/2590 (2.4%)	10/523 (1.9%)	0.54
Mycotic aneurysm	58/3113 (1.9%)	48/2590 (1.9%)	10/523 (1.9%)	0.93
Acute renal failure	548/3113 (17.6%)	441/2590 (17.0%)	107/523 (20.5%)	0.06
Persistent fever	350/2837 (12.3%)	287/2337 (12.3%)	63/500 (12.6%)	0.84
Increasing vegetation size	201/3113 (6.5%)	170/2590 (6.6%)	31/523 (5.9%)	0.59
New abscess	193/3113 (6.2%)	174/2590 (6.7%)	19/523 (3.6%)	0.008
AV block	128/2837 (4.5%)	111/2337 (4.7%)	17/500 (3.4%)	0.19
Thrombopenia	214/2837 (7.5%)	181/2337 (7.7%)	33/500 (6.6%)	0.38
Heart failure	370/2579 (14.3%)	295/2161 (13.7%)	75/418 (17.9%)	0.02
Valve or prosthetic dysfunction	439/2582 (17.0%)	352/2164 (16.3%)	87/418 (20.8%)	0.02
Length of stay	42.1 (±39.9)	42.3 (±38.7)	41.1 (±45.6)	0.03
Death at 30 days	341/3113 (11.0%)	263/2590 (10.2%)	78/523 (14.9%)	0.001
In-hospital death	529/3113 (17.0%)	424/2590 (16.4%)	105/523 (20.1%)	0.04
**Recurrence of IE at 1 year**				
Overall	57/1605 (3.6%)	51/1341 (3.8%)	6/264 (2.3%)	0.22
Medical therapy	30/742 (4.0%)	28/621 (4.5%)	2/121 (1.7%)	0.21
Surgical therapy	27/859 (3.1%)	23/718 (3.2%)	4/141 (2.8%)	1.00
**NYHA functional Classes III and IV at follow-up**				
Overall	129/1567 (8.2%)	111/1319 (8.4%)	18/248 (7.3%)	0.54
Medical therapy	74/716 (10.3%)	63/602 (10.5%)	11/114 (9.6%)	0.79
Surgical therapy	55/847 (6.5%)	48/716 (6.7%)	7/131 (5.3%)	0.56
**Surgical procedures after the index hospitalization**				
Overall	153/3113 (4.9%)	130/2590 (5.0%)	23/523 (4.4%)	0.55
Medical therapy	87/1608 (5.4%)	71/1322 (5.4%)	16/286 (5.6%)	0.88
Surgical therapy	66/1488 (4.4%)	59/1259 (4.7%)	7/229 (3.1%)	0.27
**Death at 1 year**				*P*-value at 1 year^a^
Overall	719/3113 (23.1%)	584/2590 (22.5%)	135/523 (25.8%)	0.04
Survival rate (standard error)	74.5% (0.8%)	75.0% (0.9%)	71.6% (2.1%)	
Medical therapy	474/1608 (29.5%)	380/1322 (28.7%)	94/286 (32.9%)	0.04
Survival rate (standard error)	67.6% (1.2%)	68.5% (1.4%)	63.5% (3.1%)	
Surgical therapy	236/1488 (15.9%)	199/1259 (15.8%)	37/229 (16.2%)	0.09
Survival rate (standard error)	96.7% (0.6%)	96.6% (0.7%)	97.1% (1.4%)	

**Figure 1:**
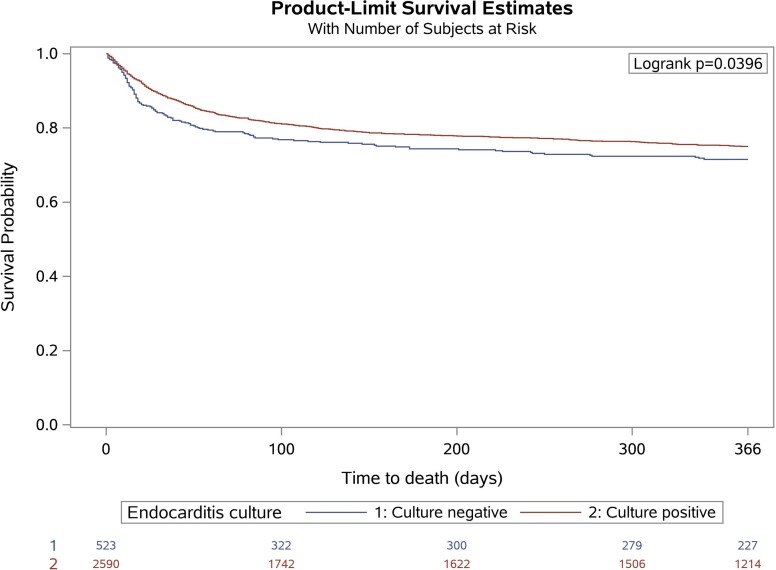
Kaplan–Meier curves demonstrating freedom from mortality during follow-up in patients with culture-positive and culture-negative endocarditis. Graphs have been plotted by EORP.

**Figure 2:**
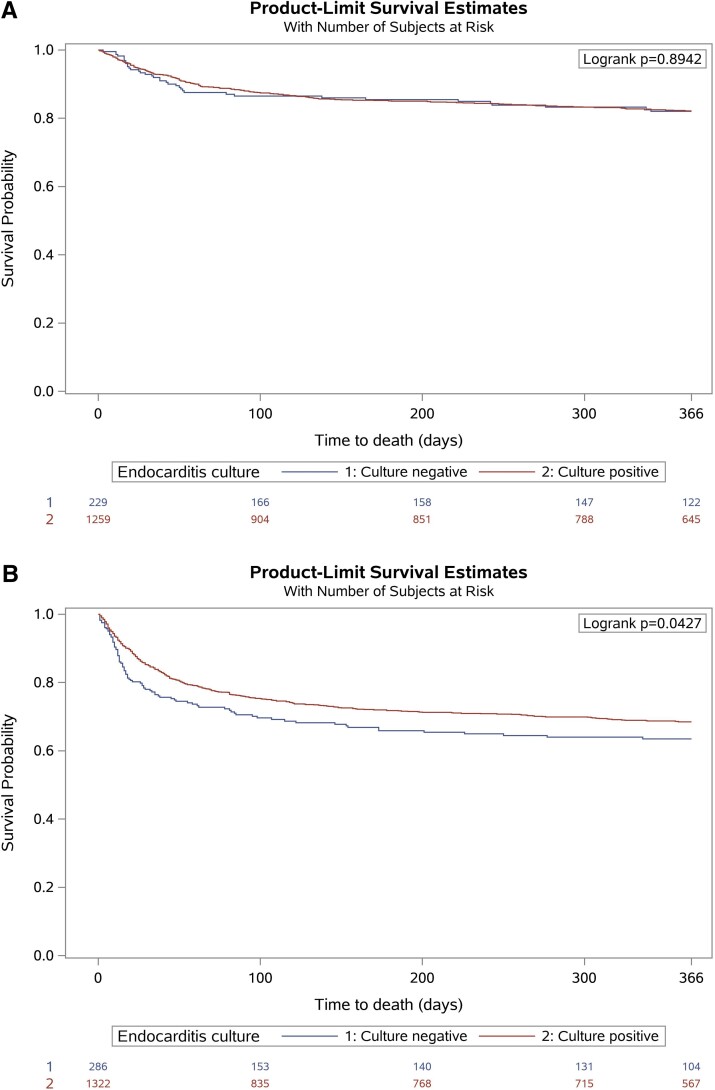
Kaplan–Meier curves demonstrating freedom from mortality during follow-up in patients with culture-positive and culture-negative endocarditis treated with (*A*) medical therapy alone and (*B*) cardiac surgery during index hospitalization.

AV, atrioventricular; CNIE, culture-negative infective endocarditis; CPIE: culture-positive infective endocarditis; IE: infective endocarditis.

a Results from actuarial survival curves: survival rate, standard error, and log-rank tests (see text and Figures 1 and 2).

The 30-day mortality was significantly higher in CNIE than CPIE patients (14.9 vs. 10.2%, *P* = 0.001; *Table 2*). The 30-day mortality was also higher in CNIE than CPIE patients in the subgroups of native valve IE [49/312 (15.7%) vs. 148/1451 (10.2%), *P* = 0.005], prosthetic valve IE [25/155 (16.1%) vs. 103/784 (13.1%), *P* = 0.32], single valve IE [53/381 (13.9%) vs. 195/1890 (10.3%), *P* = 0.04], multiple valve IE [21/86 (24.4%) vs. 53/345 (15.4%), *P* = 0.05], and isolated implantable cardioverter defibrillator/pacemaker IE [7/50 (14.0%) vs. 17/256 (6.6%), *P* = 0.09].

In the subgroup of patients who underwent surgery during the index hospitalization, the 30-day postoperative mortality was 6.3% (93/1488 patients), and it was 6.1% (77/1259 patients) in operated patients with CPIE vs. 7% (16/229) in operated patients with CNIE (*P* = 0.61).

The results of univariable and multivariable analyses of factors associated with 30-day mortality are reported in Supplementary material online, *Table S4* and in *Table 3*, respectively. At multivariable analysis, chronic heart failure [HR 1.69, 95% confidence interval (CI) 1.32–2.17], cerebral embolism (HR 1.59, 95% CI 1.16–2.18), congestive heart failure (HR 1.35, 95% CI 1.04–1.74) or electrocardiogram (ECG) conduction abnormality on admission (HR for third-degree AV block: 2.64, 95% CI 1.55–4.47), aortic (HR 1.65, 95% CI 1.30–2.11) or mitral site of IE (HR 1.69, 95% CI 1.33–2.15), cardiogenic shock (HR 1.96, 95% CI 1.43–2.67), cerebral haemorrhage (HR 2.18, 95% CI 1.32–3.61), embolic events (HR 1.83, 95% CI 1.41–2.38), and septic shock (HR 2.97, 95% CI 2.24–3.93) were significantly associated with increased 30-day mortality. On the other hand, use of MRI (HR 0.43, 95% CI 0.28–0.65) and FDG-PET scan imaging (HR 0.38, 95% CI 0.22–0.63), and surgery (HR 0.33, 95% CI: 0.25–0.43) were significantly associated with reduced 30-day mortality. As shown in *Table 3*, the HR of CNIE for 30-day mortality in multivariable analysis was 1.27 (95% CI 0.97–1.66).

**Table 3:** Multivariable Cox proportional hazard models for prediction of 30-day and 1-year mortality

	30-day mortality		1-year mortality	
Variable	Hazard ratio (95% CI)^a^	*P*-value*	Hazard ratio (95% CI)^b^	*P*-value*
Surgery	0.33 (0.25–0.43)	<0.001	0.49 (0.41–0.58)	<0.001
Chronic heart failure	1.69 (1.32–2.17)	<0.001	1.62 (1.36–1.92)	<0.001
Chronic renal failure	—	—	1.51 (1.25–1.82)	<0.001
Diabetes	—	—	1.22 (1.02–1.46)	0.03
**Symptoms**				
Cerebral embolism	1.59 (1.16–2.18)	0.004	—	—
Congestive heart failure	1.35 (1.04–1.74)	0.025	—	—
Conduction abnormality on ECG (ref: no AV block)		<0.001		<0.001
1st-degree AV block	0.71 (0.43–1.16)		0.60 (0.43–0.84)	
2nd-degree AV block	3.68 (1.16–1.66)		2.77 (1.37–5.61)	
3rd-degree AV block	2.64 (1.55–4.47)		1.65 (1.12–2.43)	
**Location of endocarditis**				
Aortic valve	1.65 (1.30–2.11)	<0.001	—	—
Mitral valve	1.69 (1.33–2.15)	<0.001	—	—
**Imaging**				
MRI	0.43 (0.28–0.65)	<0.001	—	—
PET scan	0.38 (0.22–0.63)	<0.001	—	—
**Adverse event**				
Congestive heart failure	1.59 (1.20–2.11)	0.001	—	—
Cardiogenic shock	1.96 (1.43–2.67)	<0.001	2.41 (1.91–3.04)	<0.001
Cerebral haemorrhage	2.18 (1.32–3.61)	0.002	2.84 (1.91–4.22)	<0.001
Embolic events	1.83 (1.41–2.38)	<0.001	—	—
Acute renal failure	—	—	1.54 (1.28–1.84)	<0.001
Septic shock	2.97 (2.24–3.93)	<0.001	3.45 (2.79–4.25)	<0.001
CNIE	1.27 (0.97–1.66)	0.08	1.28 (1.04–1.56)	0.02

AV, atrioventricular; CI, confidence interval; CNIE, culture negative infective endocarditis; ECG, electrocardiogram; MRI, magnetic resonance imaging; PET, positron emission tomography.

a Sample size: *N* = 2600/3113 (83.5%). Goodness of fit test: *P* = 0. 02; Concordance = 0.82; Global Schoenfeld residual test *P* = 0.67.

b Sample size: *N* = 2639/3113 (84.8%). Goodness of fit test: *P* < 0.001; Concordance = 0.75; Global Schoenfeld residual test *P* = 0.02.

* *P*-value corresponds to the results of Wald test.

The mean follow-up after discharge was 13.6 months (95% CI 13.5–13.8 months). Patients with CNIE and CPIE had comparable rates of IE recurrence and NYHA functional Classes II–IV at follow-up and need of valvular surgery after the index hospitalization (*Table 2*).

Overall, 1-year survival in the study cohort is reported in *Table 2* and shown in *Figure 1*, and factors associated with 1-year mortality in univariable and multivariable analyses are presented in Supplementary material online, *Table S5*, respectively. The CNIE (HR 1.28, 95% CI 1.04–1.56), chronic heart failure (HR 1.62, 95% CI 1.36–1.92), chronic renal failure (HR 1.51, 95% CI 1.25–1.82), diabetes (HR 1.22, 95% CI 1.02–1.46), and ECG conduction abnormality on admission (HR for third-degree AV block: 1.65, 95% CI 1.12–2.43), and the following adverse events: cardiogenic shock (HR 2.41, 95% CI 1.91-3.04), cerebral haemorrhage (HR 2.84, 95% CI 1.91–4.22), acute renal failure (HR 1.54, 95% CI 1.28–1.84), and septic shock (HR 3.45, 95% CI 2.79–4.25) were significantly associated with higher 1-year mortality at multivariable analysis. Conversely, surgery (HR 0.49, 95% CI 0.41–0.58) was significantly associated with reduced 1-year mortality.

Some differences with respect to the primary study outcomes were observed after stratification according to the use of surgical and medical therapy. Among patients with CPIE, 30-day mortality was 13.9% (184/1322) and 6.1% (77/1259) in medical and surgical patients, respectively (*P* < 0.001). Among patients with CNIE, 30-day mortality was 20.3% (58/286) and 7.0% (16/229) in medical and surgical patients, respectively (*P* < 0.001). Overall, 69.4% (1797/2590) of patients with CPIE and 69.0% (360/522) with CNIE had theoretical indications for surgery. Despite these indications, surgery was not performed in 24.8% (445/1797) of the CPIE group and 32.2% (116/360) of the CNIE group (*P* = 0.003; see Supplementary material online online, *Table S6*).

The 1 year mortality was significantly higher in CNIE than CPIE patients in the medical subgroup (*Figure 2A*) but not statistically different between CNIE and CPIE in the surgical subgroup (*Figure 2B*).

The results of sensitivity analyses on mortality are reported in Supplementary material online, *Table S7*. Briefly, Cox regression models with LASSO penalty for variable selection showed that CNIE were significantly associated with mortality both at 30 days (differently from the main study analysis) and at 1 year (as in the main study analysis). The negative impact of CNIE on 30-day and 1-year mortality was observed both in medically treated and surgically treated patients. Surgery was protective for both 30-day and 1-year mortality (see Supplementary material online, *Table S7*).

Compared with patients with *S. aureus* CPIE, CNIE patients had less embolic events (15.3 vs. 26.3%, *P* < 0.001), spondylitis (1.3 vs. 5.9%, *P* < 0.001), septic shock (7.8 vs. 15.5%, *P* < 0.001), cerebral haemorrhage (1.9 vs. 4.2%, *P* = 0.02), persistent fever (12.6 vs. 17.6%, *P* = 0.02), new abscess (3.6 vs. 6.9%, *P* = 0.01), and thrombocytopenia (6.6 vs. 9.8%, *P* = 0.05). In addition, they had more frequently valve or prosthesis dysfunction (20.8 vs. 14.8%, *P* = 0.01) at discharge. The 30-day and 1-year mortality were not significantly different in *S. aureus* IE and CNIE patients (13.8 vs. 14.9%, *P* = 0.56 at 30 days; 25.8 vs. 28.8%, *P* = 0.23 at 1 year; see Supplementary material online, *Table S8*).

## Discussion

The main results stemming from the present analysis of the EURO-ENDO registry data are the following: (i) about 1/5 patients with IE had CNIE; (ii) clinical characteristics at admission for IE were different between CNIE and CPIE patients; (iii) 30-day mortality was higher in CNIE than CPIE patients; (iv) among patients with theoretical indications for surgery, patients with CNIE were less frequently operated upon than those with CPIE; (v) no difference in 1-year survival was observed between CNIE and CPIE in the subgroup of surgically treated patients, but a lower survival for CNIE was observed in patients receiving medical treatment alone (*Structured Graphical Abstract*).

### Culture-negative infective endocarditis: prevalence and diagnosis

Among patients with IE enrolled in the EURO-ENDO registry, 16.8% had CNIE. This is among the lowest registered frequencies of CNIE when compared with other series (where frequency ranges from 9 to 42%), possibly testifying to improved approaches to the aetiological diagnosis of IE over the years (especially in ESC-affiliated countries which enrolled the majority of patients in the registry), although the reasons may be more complex and multifactorial.^5–11^ Indeed, it should be remembered that CNIE is a heterogeneous condition including different types of diseases, some rare (i.e. marantic endocarditis, endocarditis related to systemic diseases, such as lupus and Behçet, IE due to fastidious microorganisms such as HACEK or nutritionally variant streptococci, IE due to intracellular bacteria not routinely cultured in blood samples), and others likely far more frequent (i.e. IE with no aetiological diagnosis because of reduced sensitivity of blood cultures collected after initiation of antimicrobial therapy).^12–14^ The latter may be in line with the slightly higher number of antibiotics received prior to admission by CNIE than CPIE patients in the present study that may have affected the diagnostic yield of cultures.

It could be of interest to explore in future studies whether heterogeneous antibiotic practices or other reasons may had been responsible for the higher prevalence of CNIE, we registered in non-ESC-affiliated than in ESC-affiliated countries.^15^

### Culture-negative infective endocarditis characteristics and outcomes

In the present cohort from the EURO-ENDO registry, CNIE patients were younger with less ischaemic heart disease, diabetes mellitus, and hypertension than patients with CPIE. Overall, baseline signs and symptoms such as fever, septic shock, and spondylitis were more frequently observed in patients with CPIE, whereas heart failure due to valvular dysfunction was more frequently observed in patients with CNIE. However, it is interesting that the difference in advanced disease (valve dysfunction, heart failure) was more clearly appreciable not at baseline, but over the disease course (*Table 2*). Notably, similar differences in patient characteristics in EURO-ENDO were also found in the GAMES study.^16^ All of this may suggest that CNIE diagnoses and/or evaluation for surgery were more frequently obtained later than in patients with CPIE, and after the occurrence of cardiac or systemic complications. On the other hand, we cannot exclude that the increased frequency of heart failure and valvular dysfunction was the consequence of, rather than the reason for, an initial conservative approach, which: (i) may cast doubts about the appropriateness of the initial conservative decision; and (ii) may support a detrimental effect on functional status of an only partly controlled septic process after an initially appropriate conservative decision, consequent of the non-identification (and lack of antibiogram) of the causative agent of CNIE, with possibly suboptimal antimicrobial therapy.^17^

The increased number of new postoperative abscesses in CPIE we found in our study has not a clear explanation and deserves further investigation, although one possible speculation is that patients with CPIE might present with initially more destructive disease, with earlier diagnosis of CPIE vs. CNIE mitigating or even reversing the unfavourable prognostic impact of destructive disease. This is also in line with the lack of difference in the prognostic impact of CNIE vs. CPIE due to *S. aureus*, since the latter are among the most destructive CPIE and are associated with high mortality.^5^

Of note, the softening effect on signs and symptoms of IE of broad spectrum antimicrobials administered prior to diagnosis/evaluation may have played a role in influencing initial surgical decisions in CNIE patients, although this hypothesis deserves further investigation by means of dedicated studies registering the precise timing of antimicrobial therapy with respect to diagnosis/evaluation.

The possible impact of CNIE on short- and long-term mortality is a current subject of debate and EURO-ENDO can provide some interesting insights. According to some series, CNIE is not associated with increased early mortality,^6,9,18^ whereas others suggest a relevant, unfavourable impact on early and/or long-term mortality.^7,11,16^ In our series, 30-day mortality was approximately 5% higher in CNIE than CPIE patients. Notably, although the direction of the effect was towards increased mortality, CNIE did not turn out to be a predictor at 30 days at stepwise multivariable analysis but was associated with 30-day mortality at LASSO regression. Although the different results of the two models certainly call for further investigation, the results of the additional LASSO sensitivity analysis are in line with the unfavourable impact of CNIE on 1-year mortality, noticed both in stepwise and LASSO models.

The GAMES study (the largest study after EURO-ENDO), whose observation period is limited to in-hospital stay, refers to CNIE as a risk factor for in-hospital mortality related to late and inappropriate antimicrobial therapy.^16^ The results of our series suggest that the higher mortality observed in the CNIE group may also rely on other indirect reasons, such as the possible diagnostic/surgical evaluation delay in CNIE patients discussed in the previous paragraph. Form this standpoint, early mortality may be associated with patient characteristics and the use of surgery, which can mitigate the effects of uncontrolled infection.

Of note, the impact of treatment modality (surgery vs. medical treatment alone) was seldom taken into account in previous series.^6,7,9,11,16,18^ In the present study, advanced disease possibly discouraging the use of surgery because of absolute/relative contraindications was more frequently observed in CNIE than CPIE patients. All of this is in line with the fact that CNIE patients with theoretical surgical indications were less frequently operated upon than CPIE patients. However, as discussed above, further studies are needed to support this hypothesis.

Finally, certainty of cure with surgery, which was significantly associated with lower mortality in multivariable analyses, and a potentially insidious infection may explain why 1-year survival was lower for CNIE in patients receiving medical treatment alone and comparable between CNIE and CPIE in surgically treated patients.

### Limitations

Our study has some limitations. The first is the observational nature of the analysis, which may imply some information and selection bias. Second, this is an ancillary analysis of a registry that was not primarily dedicated to the assessment of the objectives of the present—for example, there is no detailed information regarding the exact timing of antimicrobial therapy with respect to surgery and IE diagnosis. Thus, the evolution of the disease in CNIE patients could be due to an untimely antibiotic therapy.

Third, standards of treatment could be different in certain centres and/or countries. Certain diagnostic approaches such as some imaging techniques may not have been employed in all centres.^19^ The PET/CT equipment was available in only 70.3% centres in ESC-affiliated countries and in 56.3% centres elsewhere and was ultimately used in 16.6% of patients (25.0% in suspected prosthetic valve IE).^2^ In addition, the lack of specific guidelines for ^18^F-FDG-PET/CT on patient preparation, image acquisitions, semi-quantitative measurements, and image interpretation may result in low sensitivity especially for native valve IE diagnosis, thereby limiting its use in such circumstances.^20^

As reported in previous studies, patients admitted to small hospitals may not easily receive transoesophageal echocardiography or other advanced examinations that allow for early diagnosis or identification of complications that could affect prognosis.^16^ The same applies to surgical treatment in centres without cardiac surgery on site. Indeed, surgery may be underused for several reasons including, among others, patient refusal, advanced age, frailty, or high operative risk.

Fourth, information about the number of patients transferred from non-surgical centres to cardiac surgical centres is missing in the registry, preventing assessment of any possible modifying effect of this factor on the prognostic impact of surgery.

Fifth, we could not evaluate the possible additional diagnostic potential of valve PCR results, since molecular tests were not available in all centres, and also the denominator of tested patients was not available (only positive PCR results were ultimately included in the registry).^21^ For this reason, and also because only valve samples or removed parts of catheters and devices could be tested for PCR, it was not possible to estimate the true impact of molecular biology in guiding antibiotic therapy. Finally, data from critically ill patients, whose informed consent to participate in the registry could not be obtained, were not included.

## Conclusion

In conclusion, the present analysis of the EURO-ENDO registry is the largest study comparing CNIE and CPIE. We found a possibly increased short-term mortality and an increased long-term mortality in patients with CNIE when compared with those with CPIE. This difference was present in patients receiving medical treatment alone and not in those who underwent surgery, with surgery being associated with reduced mortality. Additional efforts are required both to improve the aetiological diagnosis of IE and early identification of CNIE cases before progression to advanced disease that may exclude the possibility of surgery.

## Supplementary material

Supplementary material is available at *European Heart Journal* online.

## Supplementary Material

ehac307_Supplementary_Data

## Data Availability

The data underlying this article are available in the article and in its online supplementary material. EURObservational Research Programme (EORP) for the European Society of Cardiology kindly provided statistical advice for this manuscript.

## Declaration of Helsinki

The authors should state that their study complies with the Declaration of Helsinki, that the locally appointed ethics committee has approved the research protocol, and that informed consent has been obtained from the subjects (or their legally authorized representative).

## Statistics and biometry

EURObservational Research Programme (EORP) for the European Society of Cardiology kindly provided statistical advice for this manuscript. C.L. was the Statistical Project Lead.

## Study subjects or cohorts overlap

Some study subjects or cohorts have been previously reported in ‘Habib G, Erba PA, Iung B, Donal E, Cosyns B, Laroche C, Popescu BA, Prendergast B, Tornos P, Sadeghpour A, Oliver L, Vaskelyte JJ, Sow R, Axler O, Maggioni AP, Lancellotti P; EURO-ENDO Investigators. Clinical presentation, aetiology and outcome of infective endocarditis. Results of the ESC-EORP EURO-ENDO (European infective endocarditis) registry: a prospective cohort study. *Eur Heart J* 2019;40(39):3222–3232. doi:10.1093/eurheartj/ehz620. Erratum in: *Eur Heart J* 2020;41(22):2091. PMID: 31504413’.

## Methodology

This study was a prospective, observational, multicentre study.

## Appendix 1

### European Infective Endocarditis registry (EURO-ENDO)

#### Executive Committee

Gilbert Habib, FR (Chair); Patrizio Lancellotti, BE (Chair); Bernard Cosyns, BE; Erwan Donal, FR; Paola Erba, IT; Gilbert Habib, FR; Bernard Iung, FR; Aldo P. Maggioni, IT; Bogdan A. Popescu, RO; Bernard Prendergast, GB; Pilar Tornos, ES

#### National Coordinators

Nora Nabila Ali Tatar-Chentir, DZ; Mouaz Al-Mallah, SA; Meriam Astrom Aneq, SE; George Athanassopoulos, GR; Luigi Paolo Badano, IT; Soraya Benyoussef, TN; Erick Calderon Aranda, MX; Nuno Miguel Cardim, PT; Kwan-Leung Chan, CA; Bernard Cosyns, BE; Ines Cruz, PT; Thor Edvardsen, NO; Georg Goliasch, AT; Gilbert Habib, FR; Andreas Hagendorff, DE; Krasimira Hristova, BG; Bernard Iung, FR; Otto Kamp, NL; Duk-Hyun Kang, KR; William Kong, SG; Simon Matskeplishvili, RU; Marwa Meshaal, EG; Maja Mirocevic, ME; Aleksandar N. Neskovic, RS; Michal Pazdernik, CZ; Edyta Plonska-Gosciniak, PL; Bogdan A. Popescu, RO; Bernard Prendergast, GB; Maha Raissouni, MA; Ricardo Ronderos, AR; Leyla Elif Sade, TR; Anita Sadeghpour, IR; Antonia Sambola, ES; Shantanu Sengupta, IN; Jadranka Separovic-Hanzevacki, HR; Masaaki Takeuchi, JP; Edwin Tucay, PH; Ana Clara Tude Rodrigues, BR; Albert Varga, HU; Jolanta Vaskelyte, LT; Kentaro Yamagata, MT; Kyriakos Yiangou, CY; Hosam Zaky, AE

#### Investigators

**Argentina:** *Buenos Aires:* R. Ronderos, G. Avegliano, P. Fernandez Oses, E. Filipini, I. Granada, A. Iribarren, M. Mahia, F. Nacinovich, S. Ressi, *Corrientes:* R. Obregon, M. Bangher, J. Dho, *La Plata:* L. Cartasegna, M.L. Plastino, V. Novas, C. Shigel, *Florencio Varela:* G. Reyes, M. De Santos, N. Gastaldello, M. Granillo Fernandez, M. Potito, G. Streitenberger, P. Velazco, *Buenos Aires:* J.H. Casabé, C. Cortes, E. Guevara, F. Salmo, M. Seijo; **Austria:***Vienna:* F. Weidinger, M. Heger, R. Brooks, C. Stöllberger, C-Y. Ho, L. Perschy, L. Puskas, *Vienna:* G. Goliasch, C. Binder, R. Rosenhek, M. Schneider, M-P. Winter; **Belgium:***Liege:* E. Hoffer, M. Melissopoulou, E. Lecoq, D. Legrand, S. Jacquet, M. Massoz, *Liege:* P. Lancellotti, L. Pierard, R. Dulgheru, S. Marchetta, C. D´Emal, C. Oury, *Jette:* B. Cosyns, S. Droogmans, D. Kerkhove, A. Motoc, D. Plein, B. Roosens, L. Soens, C. Weytjens, I. Lemoine, *Edegem:* I. Rodrigus, B. Paelinck, B. Amsel, *Brussels:* P. Unger, D. Konopnicki, *Brussels:* C. Beauloye, A. Pasquet, S. Pierard, D. Vancraeynest, J.L. Vanoverschelde, F. Sinnaeve; **Brazil:***Sao Paulo:* J.L. Andrade, A.C. Tude Rodrigues, K. Staszko, *Porto Alegre:* R. Dos Santos Monteiro, M.H. Miglioranza, D.L. Shuha, *Rio de Janeiro:* M. Alcantara, V. Cravo, L. Fazzio, A. Felix, M. Iso, C. Musa, A.P. Siciliano, *Marilia:* F. Villaca Filho, J. Braga, A. Rodrigues, R. Silva, F. Vilela, D. Rodrigues, L. Silva, *Sao Paulo:* S. Morhy, C. Fischer, R. Silva, M. Vieira, T. Afonso, *Fortaleza:* J. Abreu, S.N. Falcao, *Sao Paulo:* V. Moises, A. Gouvea, G. João, F. Mancuso, C. Silva, A.C. Souza, *Sao Paulo:* C.S. Abboud, R. Bellio de Mattos Barretto, A. Ramos, R. Arnoni, J.E. Assef, D.J. Della Togna, D. Le Bihan, L. Miglioli, A.P. Romero Oliveira, R. Tadeu Magro Kroll, D. Cortez, *Belo Horizonte:* C.L. Gelape, M.d.C. Peirira Nunes, T.C. De Abreu Ferrari; **Canada:***Ottawa:* K-L. Chan, K. Hay, *Montreal:* V. Le, M. Page, F. Poulin, C. Sauve, K. Serri, C. Mercure, *Quebec:* J. Beaudoin, P. Pibarot, *Montreal:* I. Sebag, L. Rudski, G. Ricafort; **Croatia:***Zagreb:* B. Barsic, V. Krajinovic, M. Vargovic, *Zagreb:* J. Separovic-Hanzevacki, D. Lovric, V. Reskovic-Luksic, *Zagreb:* J. Vincelj, S. Jaksic Jurinjak; **Cyprus:***Nicosia:* V. Yiannikourides, M. Ioannides, C. Kyriakou, C. Pofaides, V. Masoura, K. Yiangou; **Czech Republic:***Ostrava-Poruba:* J. Pudich, *Prague:* A. Linhart, M. Siranec, J. Marek, *Prague:* K. Blechova, M. Kamenik, *Prague:* M. Pazdernik, *Hradec Kralove:* R. Pelouch, *Zlin:* Z. Coufal, M. Mikulica, M. Griva, E. Jancova, M. Mikulcova, *Olomouc:* M. Taborsky, J. Precek, M. Jecmenova, J. Latal, *Liberec:* J. Widimsky, *Prague:* T. Butta, S. Machacek, *Pilsen:* R. Vancata, *Brno:* J. Spinar, M. Holicka; **Ecuador:***Guayaquil:* F. Pow Chon Long, N. Anzules, A. Bajana Carpio, G. Largacha, E. Penaherrera, D. Moreira; **Egypt:***Mansoura:* E. Mahfouz, E. Elsafty, A. Soliman, Y. Zayed, J. Aboulenein, *Alexandria:* M. Abdel-Hay, A. Almaghraby, M. Abdelnaby, M. Ahmed, B. Hammad, Y. Saleh, H. Zahran, O. Elgebaly, *Zagazig:* A. Saad, M. Ali, *Alexandria:* A. Zeid, R. El Sharkawy, *Cairo:* M. Meshaal, A. Al Kholy, R. Doss, D. Osama, H. Rizk, A. Elmogy, M. Mishriky; **France:***Kremlin-Bicêtre:* P. Assayag, S. El Hatimi, *Saint-Etienne:* E. Botelho-Nevers, S. Campisi, J-F. Fuzellier, A. Gagneux-Brunon, R. Pierrard, C. Tulane, M. Detoc, T. Mehalla, *Nantes:* D. Boutoille, O. Al Habash, N. Asseray-Madani, C. Biron, J. Brochard, J. Caillon, C. Cueff, T. Le Tourneau, A.S. Lecompte, R. Lecomte, M. Lefebvre, M.M. Magali Michel, S. Pattier, S. Delarue, M. Le Bras, J. Orain, *Limoges:* J-F. Faucher, V. Aboyans, A. Beeharry, H. Durox, M. Lacoste, J. Magne, D. Mohty, A. David, V. Pradel, *Thonon-les-Bains:* V. Sierra, A. Neykova, B. Bettayeb, S. Elkentaoui, B. Tzvetkov, G. Landry, *Reims:* C. Strady, K. Ainine, S. Baumard, C. Brasselet, C. Tassigny, V. Valente-Pires, M. Lefranc, *Pointe-à-Pitre:* B. Hoen, B. Lefevre, E. Curlier, C. Callier, N. Fourcade, *Brest:* Y. Jobic, S. Ansard, R. Le Berre, P. Le Roux, F. Le Ven, M-C. Pouliquen, G. Prat, *Rouen:* F. Bouchart, A. Savoure, C. Alarcon, C. Chapuzet, I. Gueit, *Amiens:* C. Tribouilloy, Y. Bohbot, F. Peugnet, M. Gun, *Paris:* B. Iung, X. Duval, X. Lescure, E. Ilic-Habensus, *Nancy:* N. Sadoul, C. Selton-Suty, F. Alla, E. Chevalier, F. Goehringer, O. Huttin, *Poitiers:* R. Garcia, V. Le Marcis, *Rennes:* P. Tattevin, E. Donal, E. Flecher, M. Revest, *Marseille:* G. Habib, S. Hubert, J-P Casalta, F. Gouriet, F. Arregle, S. Cammilleri, L. Tessonnier, A. Riberi, *Besançon:* C. Chirouze, K. Bouiller, A-S. Brunel, D. Fournier, L. Hustache-Mathieu, T. Klopfenstein, J. Moreau, *Créteil:* P. Lim, L. Oliver, J. Ternacle, A. Moussafeur, *Dijon:* P. Chavanet, L. Piroth, M. Buisson, S. Mahy, C. Martins, A. Salmon-Rousseau, S. Gohier; **Germany:***Bad Oeynhausen:* C. Piper, J. Börgermann, D. Guckel, D. Horstkotte, B. Brockmeier, E. Winkelmann, *Leipzig:* A. Hagendorff, D. Grey, *Bonn:* G. Nickenig, R. Schueler, C. Öztürk, E. Stöhr, *Bad Nauheim:* C. Hamm, T. Walther, R. Brandt, A-C. Frühauf, C.T. Hartung, C. Hellner, C. Wild, *Aachen:* M. Becker, S. Hamada, W. Kaestner, *Berlin:* K. Stangl, F. Knebel, G. Baldenhofer, A. Brecht, H. Dreger, C. Isner, F. Pfafflin, M. Stegemann, *Ludwigshafen:* R. Zahn, B. Fraiture, C. Kilkowski, A-K. Karcher, S. Klinger, H. Tolksdorf; **Greece:***Athens:* D. Tousoulis, C. Aggeli, G. Sarri, S. Sideris, E. Venieri, *Athens:* G. Athanassopoulos, D. Tsiapras, I. Armenis, A. Koutsiari, *Athens:* G. Floros, C. Grassos, S. Dragasis, *Athens:* L. Rallidis, C. Varlamos, *Ioannina:* L. Michalis, K. Naka, A. Bechlioulis, A. Kotsia, L. Lakkas, K. Pappas, *Athens:* C. Papadopoulos, S. Kiokas, A. Lioni, S. Misailidou, *Athens:* J. Barbetseas, M. Bonou, C. Kapelios, I. Tomprou, K. Zerva, *Voula:* A. Manolis, E. Hamodraka, D. Athanasiou, G. Haralambidis, L. Poulimenos, H. Samaras; **Hungary:***Budapest:* A. Nagy, A. Bartykowszki, E. Gara; **India:***Nagpur:* S. Sengupta, K. Mungulmare, *Gurgaon:* R. Kasliwal, M. Bansal, A. Bhan, S. Ranjan; **Iran:***Tehran:* M. Kyavar, M. Maleki, F. Noohi Bezanjani, A. Sadeghpour, A. Alizadehasl, S. Boudagh, A. Ghavidel, P. Moradnejad, H.R. Pasha, B. Ghadrdoost; **Israel:***Jerusalem:* D. Gilon, J. Strahilevitz, S. Israel, M. Wanounou; **Italy:***Bari:* C. d'Agostino, P. Colonna, L. De Michele, F. Fumarola, M. Stante, *Florence:* N. Marchionni, V. Scheggi, B. Alterini, S. Del Pace, P. Stefano, C. Sparano, *Padova:* L.P. Badano, D. Muraru, N. Ruozi, R. Tenaglia, *Grosseto:* U. Limbruno, A. Cresti, P. Baratta, M. Solari, *Milan:* C. Giannattasio, A. Moreo, B. De Chiara, B. Lopez Montero, F. Musca, C.A. Orcese, F. Panzeri, C.F. Russo, F. Spano, *Milan:* O. Alfieri, M. De Bonis, E. Agricola, E. Busnardo, S. Carletti, B. Castiglioni, S. Chiappetta, B. Del Forno, D. Ferrara, M. Guffanti, G. Iaci, E. Lapenna, T. Nisi, C. Oltolini, U. Pajoro, R. Pasciuta, M. Ripa, P. Scarpellini, C. Tassan Din, R. Meneghin, D. Schiavi, *Salerno:* F. Piscione, R. Citro, R.M. Benvenga, L. Greco, C. Prota, I. Radano, L. Soriente, M. Bellino, D. Di Vece, *Genoa:* F. Santini, A. Salsano, G.M. Olivieri, *Modena:* F. Turrini, R. Messora, *Modena:* S. Tondi, A. Olaru, V. Agnoletto, L. Grassi, C. Leonardi, S. Sansoni, *Turin:* S. Del Ponte, G.M. Actis Dato, A. De Martino; **Japan:***Nagoya:* N. Ohte, S. Kikuchi, K. Wakami, *Tsukuba:* K. Aonuma, Y. Seo, T. Ishizu, T. Machino-Ohtsuka, M. Yamamoto, N. Iida, H. Nakajima, *Tenri:* Y. Nakagawa, C. Izumi, M. Amano, M. Miyake, K. Takahashi, *Osaka:* I. Shiojima, Y. Miyasaka, H. Maeba, Y. Suwa, N. Taniguchi, S. Tsujimoto, *Kobe:* T. Kitai, M. Ota, *Sapporo:* S. Yuda, S. Sasaki, *Tokyo:* N. Hagiwara, K. Yamazaki, K. Ashihara, K. Arai, C. Saitou, S. Saitou, G. Suzuki, *Miyazaki:* Y. Shibata, N. Watanabe, S. Nishino, K. Ashikaga, N. Kuriyama, *Tokyo:* K. Mahara, K. Abe, H. Fujimaki, T. Okubo, H. Shitan, S. Takanashi, M. Terada, H. Yamamoto, *Tokushima:* M. Sata, H. Yamada, K. Kusunose, Y. Saijo, H. Seno, O. Yuichiro, *Suita:* Y. Sakata, H. Mizuno, S. Nakatani, T. Onishi, K. Sengoku, F. Sera; **Korea, Republic Of:***Seoul:* S.W. Park, K. Eun Kyoung, L. Ga Yeon, J-w. Hwang, C. Jin-Oh, S-J. Park, L. Sang-Chol, C. Sung-A, S.Y. Jang, *Seoul:* D-H. Kang, R. Heo, S. Lee, J-M. Song, E. Jung; **Lithuania:***Siauliai:* J. Plisiene, A. Dambrauskaite, G. Gruodyte, *Kaunas:* R. Jonkaitiene, J. Vaskelyte, V. Mizariene, J. Atkocaityte, R. Zvirblyte; **Luxembourg:***Luxembourg:* R. Sow, A. Codreanu, E.C.L. De la Vega, C. Michaux, T. Staub, L. Jacobs-Orazi; **Malta:***Msida:* C. Mallia Azzopardi, R.G. Xuereb, T. Piscopo, D. Borg, R. Casha, J. Farrugia, M. Fenech, E. Pllaha, C. Vella, K. Yamagata; **Moldova, Republic Of:***Chisinau:* L. Grib, E. Raevschi, A. Grejdieru, G. Balan, I. Cardaniuc, L. Cardaniuc, V. Corcea, A. Feodorovici, V. Gaina, L. Girbu, P. Jimbei, D. Kravcenco, E. Panfile, E. Prisacari, E. Samohvalov, S. Samohvalov, N. Sceglova, I. Benesco, V. Marian, N. Sumarga; **Montenegro:***Podgorica:* M. Mirocevic, B. Bozovic, N. Bulatovic, P. Lakovic, L. Music; **Netherlands:***Rotterdam:* J. Roos-Hesselink, R. Budde, T. Gamela, A. Wahadat, *Amsterdam:* O. Kamp, T. Meijers, *Groningen:* J.P. Van Melle, V.M. Deursen, *Maastricht:* H. Crijns, S. Bekkers, E. Cheriex, M. Gilbers, B. Kietselaer, C. Knackstedt, R. Lorusso, S. Schalla, S. Streukens, *Utrecht:* S. Chamuleau, M-J. Cramer, A. Teske, T. Van der Spoel, A. Wind, O. Liesbek, J. Lokhorst, H. Van Heusden, *The Hague:* W. Tanis, I. Van der Bilt, J. Vriend, H. De Lange-van Bruggen, E. Karijodikoro, *Amsterdam:* R. Riezebos, E. van Dongen, J. Schoep, V. Stolk; **New Caledonia:***Noumea:* O. Axler, F. Baumann, S. Lebras; **Norway:***Oslo:* T. Edvardsen, J.T. Offstad, J.O. Beitnes, T. Helle-Valle, H. Skulstad, R. Skardal; **Pakistan:***Karachi:* N. Qamar, S. Furnaz, B. Ahmed, M.H. Butt, M.F. Khanzada, T. Saghir, A. Wahid; **Poland:***Warsaw:* T. Hryniewiecki, P. Szymanski, K. Marzec, M. Misztal-Ogonowska, *Wroclaw:* W. Kosmala, M. Przewlocka-Kosmala, A. Rojek, K. Woznicka, J. Zachwyc, *Bialystok:* A. Lisowska, M. Kaminska, *Lodz:* J. Kasprzak, E. Kowalczyk, D.F. Strzecka, P. Wejner-Mik; **Portugal:***Carnaxide:* M. Trabulo, P. Freitas, S. Ranchordas, G. Rodrigues, *Guilhufe:* P. Pinto, C. Queiros, J. Azevedo, L. Marques, D. Seabra, *Lisbon:* L. Branco, J. Abreu, M. Cruz, A. Galrinho, R. Moreira, P. Rio, A.T. Timoteo, M. Selas, *Lisbon:* N.M. Cardim, V. Carmelo, B. Duque Neves, *Almada:* H. Pereira, I. Cruz, A. Guerra, A. Marques, I. Pintassilgo; **Romania:***Timisoara:* M.C. Tomescu, N-M. Trofenciuc, M. Andor, A. Bordejevic, H.S. Branea, F. Caruntu, L. Cirin, I.M. Citu, C.A. Cotoraci, D. Darabantiu, R. Farcas, I. Marincu, A. Mavrea, M.F. Onel, T. Parvanescu, D. Pop, A.L. Pop-Moldovan, M.I. Puticiu, L.A. Velcean, *Timisoara:* A. Ionac, D. Cozma, C. Mornos, F. Goanta, I. Popescu, *Cluj-Napoca:* R. Beyer, R. Mada, R. Rancea, H. Rosianu, R. Tomoaia, C. Stanescu; **Russian Federation:***Moscow:* Z. Kobalava, J. Karaulova, E. Kotova, A. Milto, A. Pisaryuk, N. Povalyaev, M. Sorokina; **Saudi Arabia:***Jeddah:* J. Alrahimi, A. Elshiekh, *Riyadh:* A. Jamiel, A. Ahmed, M. Al-Mallah, N. Attia; **Serbia:***Belgrade:* B. Putnikovic, A. Neskovic, A. Dimic, *Belgrade:* B. Ivanovic, S. Matic, D. Trifunovic, J. Petrovic, *Belgrade:* D. Kosevic, P. Dabic, P. Milojevic, I. Petrovic, I. Stojanovic, *Sremska Kamenica:* I. Srdanovic, M. Kovacevic, A. Redzek, M. Stefanovic, S. Susak, L. Velicki, A. Vulin; **Singapore:***Singapore:* T.C. Yeo, W. KF Kong, K.K. Poh; **Spain:***Madrid:* I. Vilacosta, M. Abd El- Nasser, C. Ferrera, C. Olmos, *Vigo - Pontevedra:* F. Calvo Iglesias, E. Blanco-Gonzalez, M. Bravo Amaro, A.N. Germinas, E. Lopez-Rodriguez, J. Lugo Adan, P. Pazos-Lopez, M. Pereira Loureiro, M.T. Perez, S. Raposeiras-Roubin, S. Rasheed Yas, M-M. Suarez-Varela, F. Vasallo Vidal, *Barcelona:* D. Garcia-Dorado, A. Sambola, N. Fernandez-Hidalgo, T. Gonzalez-Alujas, J. Lozano, O. Maisterra, N. Pizzi, R. Rios, P. Tornos, *Badalona:* A. Bayes-Genis, L. Pedro Botet, N. Vallejo, E. Berastegui, C. Llibre, L. Mateu, R. Nunez, D. Quesada, *Girona:* D. Bosch Portell, J. Aboal Vinas, X. Albert Bertran, R. Brugada Tarradellas, P. Loma-Osorio Ricon, C. Tiron de Llano, *Valencia:* M.A. Arnau, A. Bel, M. Blanes, A. Osa, *Cordoba:* M. Anguita, F. Carrasco, J. Castillo, *Madrid:* J.L. Zamorano, J.L. Moya Mur, M. Alvaro, C. Fernandez-Golfin, J.M. Monteagudo, E. Navas Elorza, *Santander:* M.C. Farinas Alvarez, J. Aguero Balbin, C. Arminanzas, F. Arnaiz de las Revillas, A. Arnaiz Garcia, M. Cobo Belaustegui, M. Fernandez Sampedro, M. Gutierrez Cuadra, J.F. Gutierrez-Diez, J. Zarauza, L. Garcia Cuello, C. Gonzalez Rico, *Barakaldo:* R. Rodriguez-Alvarez, J. Goikoetxea, M. Montejo, *Barcelona:* J. Miro, M. Almela, J. Ambrosioni, C. Falces, D. Fuster, C. Garcia-de-la-Maria, M. Hernandez-Meneses, J. Llopis, F. Marco, A. Moreno, E. Quintana, E. Sandoval, A. Tellez, J.M. Tolosana, B. Vidal, I. Ruiz-Zamora, *Tarragona:* A. Bardaji Ruiz, E. Sanz Girgas, G. Garcia-Pardo, M. Guillen Marzo, A. Rodriguez Oviedo, A. Villares Jimenez; **Tunisia:***Sfax:* L. Abid, R. Hammami, S. Kammoun, *Tunis:* M.S. Mourali, F. Mghaieth Zghal, M. Ben Hlima, S. Boudiche, S. Ouali, *La Marsa:* L. Zakhama, S. Antit, I. Slama; **Turkey:***Samsun:* O. Gulel, M. Sahin, *Ankara:* L.E. Sade, E. Karacaglar, *Istanbul:* S. Kucukoglu, O. Cetinarslan, U.S. Yasar, *Ankara:* U. Canpolat, *Istanbul:* B. Mutlu, H. Atas, R. Dervishova, C. Ileri; **United Arab Emirates:***Dubai:* H. Zaky, J. Alhashmi, F. Baslib, J. Tahir, P. Zarger; **United Kingdom:***London:* S. Woldman, L. Menezes, C. Primus, R. Uppal, I. Bvekerwa, *Swindon:* B. Chandrasekaran, A. Kopanska, *London:* B. Prendergast, S. Cannata, J. Chambers, J. Hancock, J. Klein, R. Rajani, M.P. Ursi, *London:* R. Dworakowski, A. Fife, J. Breeze, M. Browne-Morgan, M. Gunning, S. Streather; **United States:***Washington:* F. Asch, M. Zemedkun; **Uzbekistan:***Tashkent:* B. Alyavi, J. Uzokov
