# Corrigendum to: Outcomes of culture-negative vs. culture-positive infective endocarditis: the ESC-EORP EURO-ENDO registry

**DOI:** 10.1093/eurheartj/ehad222

**Published:** 2023-04-13

This is a corrigendum to: William K F Kong, Antonio Salsano, Daniele Roberto Giacobbe, Bogdan A Popescu, Cécile Laroche, Xavier Duval, Robert Schueler, Antonella Moreo, Paolo Colonna, Cornelia Piper, Francisco Calvo-Iglesias, Luigi P Badano, Ilija Srdanovic, David Boutoille, Olivier Huttin, Elisabeth Stöhr, Ana Teresa Timóteo, Jolanta Justina Vaskelyte, Anita Sadeghpour, Pilar Tornos, Leila Abid, Kian Keong Poh, Gilbert Habib, Patrizio Lancellotti, on behalf of the EURO-ENDO Investigators, Outcomes of culture-negative vs. culture-positive infective endocarditis: the ESC-EORP EURO-ENDO registry, *European Heart Journal*, Volume 43, Issue 29, 1 August 2022, Pages 2770–2780, https://doi.org/10.1093/eurheartj/ehac307

In the originally published version, named members of the *EURO-ENDO Investigators* were mistakenly included in the supplementary data section, rather than the main body of the manuscript.

This has now been corrected online.

