# Supplementary material for: Outcomes of culture-negative vs. culture-positive infective endocarditis: the ESC-EORP EURO-ENDO registry
Source: Eur Heart J. 2022 Jun 8;43(29):2770–80. doi: 10.1093/eurheartj/ehac307 (PMC9459867; doi:10.1093/eurheartj/ehac307)
Supplement: ehac307_Supplementary_Data [file ehac307_supplementary_data.docx]

**Supplementary table 1.** ESC 2015 diagnostic criteria (eCRF data) in culture positive vs culture negative endocarditis

| Variable | | Total  N=3113 | CPIE  N=2590 (83.2%) | CNIE  N=523 (16.8%) | P-Value |
| --- | --- | --- | --- | --- | --- |
| Pathologic criteria | | 971/3113 (31.2%) | 869/2590 (33.6%) | 102/523 (19.5%) | <0.001 |
| Imaging positive for IE | | 2889/3113 (92.8%) | 2377/2590 (91.8%) | 512/523 (97.9%) | <0.001 |
| Predisposition | | 1934/3113 (62.1%) | 1588/2590 (61.3%) | 346/523 (66.2%) | 0.04 |
| Fever > 38°C | | 2471/3113 (79.4%) | 2057/2590 (79.4%) | 414/523 (79.2%) | 0.89 |
| Vascular phenomena | | 1050/3113 (33.7%) | 887/2590 (34.2%) | 163/523 (31.2%) | 0.17 |
| Immunological phenomena | | 375/3113 (12.0%) | 299/2590 (11.5%) | 76/523 (14.5%) | 0.06 |
| Microbiological evidence | | 212/3113 (6.8%) | 209/2590 (8.1%) | 3/523 (0.6%) | <0.001 |
| Score - Pathological criteria | | 971/3113 (31.2%) | 869/2590 (33.6%) | 102/523 (19.5%) | <0.001 |
| Score - Major criteria | 0 | 33/3113 (1.1%) | 23/2590 (0.9%) | 10/523 (1.9%) | <0.001 |
|  | 1 | 972/3113 (31.2%) | 461/2590 (17.8%) | 511/523 (97.7%) |  |
|  | 2 | 2108/3113 (67.7%) | 2106/2590 (81.3%) | 2/523 (0.4%) |  |
| Score - Minor criteria | 0 | 137/3113 (4.4%) | 125/2590 (4.8%) | 12/523 (2.3%) | 0.02 |
|  | 1 | 817/3113 (26.2%) | 661/2590 (25.5%) | 156/523 (29.8%) |  |
|  | 2 | 1373/3113 (44.1%) | 1138/2590 (43.9%) | 235/523 (44.9%) |  |
|  | 3 | 665/3113 (21.4%) | 561/2590 (21.7%) | 104/523 (19.9%) |  |
|  | 4 | 121/3113 (3.9%) | 105/2590 (4.1%) | 16/523 (3.1%) |  |
| Infective endocarditis | Definite IE | 2607/3113 (83.7%) | 2401/2590 (92.7%) | 206/523 (39.4%) | <0.001 |
|  | Possible IE | 506/3113 (16.3%) | 189/2590 (7.3%) | 317/523 (60.6%) |  |
| Definite IE | Pathological criteria | 971/2607 (37.2%) | 869/2401 (36.2%) | 102/206 (49.5%) | <0.001 |
|  | 2 major criteria | 1408/2607 (54.0%) | 1406/2401 (58.6%) | 2/206 (1.0%) |  |
|  | 1 major criteria and at least 3 minor criteria | 228/2607 (8.7%) | 126/2401 (5.2%) | 102/206 (49.5%) |  |
| Possible IE | 1 major criteria and 1 or 2 minor criteria | 492/506 (97.2%) | 180/189 (95.2%) | 312/317 (98.4%) | 0.03 |
|  | At least 3 minor criteria | 14/506 (2.8%) | 9/189 (4.8%) | 5/317 (1.6%) |  |

IE: infective endocarditis; CNIE: culture negative infective endocarditis; CPIE: culture positive infective endocarditis.

**Supplementary table 2.** Signs, symptoms and surgical details of patients with culture positive vs culture negative infective endocarditis

| **Variable** | | | **Total N=3113** | **CPIE N=2590 (83.2%)** | **CNIE N=523 (16.8%)** | **P-Value** |
| --- | --- | --- | --- | --- | --- | --- |
| *Signs and symptoms* | | | | | | |
| Fever | | | 819/2669 (30.7%) | 693/2198 (31.5%) | 126/471 (26.8%) | 0.04 |
| Cardiac murmur | | | 2005/3109 (64.5%) | 1638/2586 (63.3%) | 367/523 (70.2%) | 0.003 |
| Congestive heart failure | | | 846/3113 (27.2%) | 684/2590 (26.4%) | 162/523 (31.0%) | 0.03 |
| Cardiogenic shock | | | 63/2837 (2.2%) | 50/2337 (2.1%) | 13/500 (2.6%) | 0.53 |
| Septic shock | | | 203/3112 (6.5%) | 180/2589 (7.0%) | 23/523 (4.4%) | 0.03 |
| Osler nodes | | | 60/3113 (1.9%) | 53/2590 (2.0%) | 7/523 (1.3%) | 0.28 |
| Janeway lesion | | | 109/3113 (3.5%) | 92/2590 (3.6%) | 17/523 (3.3%) | 0.73 |
| Roth spots | | | 44/3049 (1.4%) | 37/2527 (1.5%) | 7/522 (1.3%) | 0.83 |
| Abscess | | | 363/3113 (11.7%) | 303/2590 (11.7%) | 60/523 (11.5%) | 0.88 |
| Spondylitis | | | 168/3113 (5.4%) | 159/2590 (6.1%) | 9/523 (1.7%) | <0.001 |
| Embolic events | | | 791/3113 (25.4%) | 660/2590 (25.5%) | 131/523 (25.0%) | 0.83 |
| Pulmonary embolism | | | 195/3113 (6.3%) | 159/2590 (6.1%) | 36/523 (6.9%) | 0.52 |
| Cerebral embolism | | | 350/3113 (11.2%) | 297/2590 (11.5%) | 53/523 (10.1%) | 0.38 |
| Splenic embolism | | | 176/3113 (5.7%) | 155/2590 (6.0%) | 21/523 (4.0%) | 0.07 |
| Coronary embolism | | | 22/3113 (0.7%) | 14/2590 (0.5%) | 8/523 (1.5%) | 0.02 |
| Renal embolism | | | 76/3113 (2.4%) | 66/2590 (2.5%) | 10/523 (1.9%) | 0.39 |
| Hepatic embolism | | | 17/3113 (0.5%) | 17/2590 (0.7%) | 0/523 (0.0%) | 0.09 |
| Peripheral embolism | | | 92/3113 (3.0%) | 69/2590 (2.7%) | 23/523 (4.4%) | 0.03 |
| Haemorrhagic Stroke | | | 67/3113 (2.2%) | 54/2590 (2.1%) | 13/523 (2.5%) | 0.56 |
| Conduction abnormality | | No | 2544/2875 (88.5%) | 2109/2384 (88.5%) | 435/491 (88.6%) | 0.90 |
|  |  | AV block I | 232/2875 (8.1%) | 194/2384 (8.1%) | 38/491 (7.7%) |  |
|  |  | AV block II | 17/2875 (0.6%) | 13/2384 (0.5%) | 4/491 (0.8%) |  |
|  |  | AV block III | 82/2875 (2.9%) | 68/2384 (2.9%) | 14/491 (2.9%) |  |
| *Location of Endocarditis and surgical details* | | | | | | |
| Location of Endocarditis – Aortic valve | | | 1514/3056 (49.5%) | 1260/2536 (49.7%) | 254/520 (48.8%) | 0.73 |
| Location of Endocarditis - ICD/PM | | | 333/3056 (10.9%) | 280/2536 (11.0%) | 53/520 (10.2%) | 0.57 |
| Location of Endocarditis – Mitral valve | | | 1284/3056 (42.0%) | 1056/2536 (41.6%) | 228/520 (43.8%) | 0.35 |
| Location of Endocarditis – Tricuspid valve | | | 349/3056 (11.4%) | 278/2536 (11.0%) | 71/520 (13.7%) | 0.08 |
| Location of Endocarditis - Pulmonary | | | 74/3056 (2.4%) | 59/2536 (2.3%) | 15/520 (2.9%) | 0.45 |
| Location of Endocarditis – Single valve IE | | | 2271/3113 (73.0%) | 1890/2590 (73.0%) | 381/523 (72.8%) | 0.95 |
| Location of Endocarditis – Multiple valve IE | | | 431/3113 (13.8%) | 345/2590 (13.3%) | 86/523 (16.4%) | 0.06 |
| Location of Endocarditis – Native valve IE | | | 1763/3113 (56.6%) | 1451/2590 (56.0%) | 312/523 (59.7%) | 0.13 |
| Location of Endocarditis – Prosthetic valve IE | | | 939/3113 (30.2%) | 784/2590 (30.3%) | 155/523 (29.6%) | 0.77 |
| Number of locations of endocarditis | | | 1.2 (±0.5) | 1.2 (±0.5) | 1.2 (±0.5) | 0.49 |
| EuroScore II (N=2632) | | | 10.9 (±14.6) | 11.0 (±14.6) | 10.6 (±14.6) | 0.27 |
| Surgery on the aortic valve | | | 870/1595 (54.5%) | 732/1352 (54.1%) | 138/243 (56.8%) | 0.44 |
| Type of surgery - Aortic valve | Bioprosthesis | | 507/870 (58.3%) | 441/732 (60.2%) | 66/138 (47.8%) | <0.001 |
|  | Homograft | | 48/870 (5.5%) | 45/732 (6.1%) | 3/138 (2.2%) |  |
|  | Mechanical prosthesis | | 295/870 (33.9%) | 228/732 (31.1%) | 67/138 (48.6%) |  |
|  | Repair | | 20/870 (2.3%) | 18/732 (2.5%) | 2/138 (1.4%) |  |
| Surgery on the mitral valve | | | 672/1595 (42.1%) | 567/1352 (41.9%) | 105/243 (43.2%) | 0.71 |
| Type of surgery - Mitral valve | Bioprosthesis | | 252/672 (37.5%) | 220/567 (38.8%) | 32/105 (30.5%) | 0.01 |
|  | Homograft | | 2/672 (0.3%) | 2/567 (0.4%) | 0/105 (0.0%) |  |
|  | Mechanical prosthesis | | 249/672 (37.1%) | 195/567 (34.4%) | 54/105 (51.4%) |  |
|  | Repair | | 169/672 (25.1%) | 150/567 (26.5%) | 19/105 (18.1%) |  |
| Surgery on the tricuspid valve | | | 177/1595 (11.1%) | 149/1352 (11.0%) | 28/243 (11.5%) | 0.82 |
| Type of surgery - Tricuspid valve | Bioprosthesis | | 48/177 (27.1%) | 44/149 (29.5%) | 4/28 (14.3%) | 0.01 |
|  | Homograft | | 0/177 (0.0%) | 0/149 (0.0%) | 0/28 (0.0%) |  |
|  | Mechanical prosthesis | | 11/177 (6.2%) | 6/149 (4.0%) | 5/28 (17.9%) |  |
|  | Repair | | 118/177 (66.7%) | 99/149 (66.4%) | 19/28 (67.9%) |  |
| Surgery on the pulmonary valve | | | 28/1595 (1.8%) | 24/1352 (1.8%) | 4/243 (1.6%) | 1.0 |
| Type of surgery - Pulmonary valve | Bioprosthesis | | 10/28 (35.7%) | 7/24 (29.2%) | 3/4 (75.0%) | 0.28 |
|  | Homograft | | 9/28 (32.1%) | 9/24 (37.5%) | 0/4 (0.0%) |  |
|  | Mechanical prosthesis | | 5/28 (17.9%) | 4/24 (16.7%) | 1/4 (25.0%) |  |
|  | Repair | | 4/28 (14.3%) | 4/24 (16.7%) | 0/4 (0.0%) |  |
| Percutaneous lead extraction | | | 126/1596 (7.9%) | 106/1352 (7.8%) | 20/244 (8.2%) | 0.85 |
| Surgical lead extraction | | | 119/1596 (7.5%) | 107/1352 (7.9%) | 12/244 (4.9%) | 0.10 |
| Percutaneous catheter extraction | | | 13/1466 (0.9%) | 12/1233 (1.0%) | 1/233 (0.4%) | 0.70 |

**Abbreviations:** AV: atrioventricular; CNIE: culture negative infective endocarditis; CPIE: culture positive infective endocarditis; ICD/PM: implantable cardioverter defibrillator/pacemaker.

**Supplementary table 3.** Antimicrobial therapies of patients with culture positive vs culture negative endocarditis

| Variable | Total  N=3113 | CPIE  N=2590 (83.2%) | CNIE  N=523 (16.8%) | P-Value |
| --- | --- | --- | --- | --- |
| *Before admission* | | | | |
| At least one anti-microbial therapy during the month preceding the admission | 942/2420 (38.9%) | 757/1990 (38.0%) | 185/430 (43.0%) | 0.06 |
| Number of Anti-Microbial therapies  (N=2420) | 0.7 (±1.1) | 0.7 (±1.1) | 0.9 (±1.2) | 0.01 |
| *During hospitalization* | | | | |
| Number of Anti-Microbial therapies (N=3100) | 3.5 (±1.9) | 3.5 (±2.0) | 3.1 (±1.5) | <0.001 |
| Penicillin | 270/3100 (8.7%) | 256/2578 (9.9%) | 14/522 (2.7%) | <0.001 |
| Ampicillin or amoxicillin (with or without beta-lactamases inhibitors) | 1167/3100 (37.6%) | 985/2578 (38.2%) | 182/522 (34.9%) | 0.15 |
| Ceftriaxone | 996/3100 (32.1%) | 860/2578 (33.4%) | 136/522 (26.1%) | 0.001 |
| Oxacillin | 420/3100 (13.5%) | 390/2578 (15.1%) | 30/522 (5.7%) | <0.001 |
| Vancomycin | 1364/3100 (44.0%) | 1080/2578 (41.9%) | 284/522 (54.4%) | <0.001 |
| Daptomycin | 352/3100 (11.4%) | 300/2578 (11.6%) | 52/522 (10.0%) | 0.27 |
| Rifampicin | 597/3100 (19.3%) | 520/2578 (20.2%) | 77/522 (14.8%) | 0.004 |
| Amphotericin B | 36/3100 (1.2%) | 33/2578 (1.3%) | 3/522 (0.6%) | 0.17 |
| Doxycyclin | 77/3100 (2.5%) | 52/2578 (2.0%) | 25/522 (4.8%) | <0.001 |
| Gentamycin | 1758/3100 (56.7%) | 1442/2578 (55.9%) | 316/522 (60.5%) | 0.05 |
| Clindamycin | 116/3100 (3.7%) | 108/2578 (4.2%) | 8/522 (1.5%) | 0.004 |
| Cotrimoxazole | 103/3100 (3.3%) | 93/2578 (3.6%) | 10/522 (1.9%) | 0.05 |
| Piperacillin/Tazobactam | 288/3100 (9.3%) | 251/2578 (9.7%) | 37/522 (7.1%) | 0.06 |
| Carbapenems | 438/3100 (14.1%) | 347/2578 (13.5%) | 91/522 (17.4%) | 0.02 |
| Cefazolin | 180/3100 (5.8%) | 164/2578 (6.4%) | 16/522 (3.1%) | 0.003 |
| Echinocandins | 52/3100 (1.7%) | 45/2578 (1.7%) | 7/522 (1.3%) | 0.51 |
| Fluconazole or Voriconazole | 87/3100 (2.8%) | 66/2578 (2.6%) | 21/522 (4.0%) | 0.06 |
| Linezolid | 146/3100 (4.7%) | 124/2578 (4.8%) | 22/522 (4.2%) | 0.56 |
| Anti-staphyloccocal penicillins other than oxacillin | 281/3100 (9.1%) | 245/2578 (9.5%) | 36/522 (6.9%) | 0.06 |
| Quinolones | 449/3100 (14.5%) | 364/2578 (14.1%) | 85/522 (16.3%) | 0.20 |
| Aminoglycosides other than gentamycin | 74/3100 (2.4%) | 66/2578 (2.6%) | 8/522 (1.5%) | 0.16 |
| Cephalosporins other than ceftriaxone and cefaxolin | 253/3100 (8.2%) | 204/2578 (7.9%) | 49/522 (9.4%) | 0.26 |
| Teicoplanin | 88/3100 (2.8%) | 68/2578 (2.6%) | 20/522 (3.8%) | 0.13 |
| Other | 273/3100 (8.8%) | 230/2578 (8.9%) | 43/522 (8.2%) | 0.61 |

CNIE: culture negative infective endocarditis; CPIE: culture positive infective endocarditis

**Supplementary table 4.** Univariable Cox proportional hazards models for mortality at 30-days.

| **Variable*** | | **Dead** | **Alive** | **Hazard Ratio(95% CI)** | **P-Value** |
| --- | --- | --- | --- | --- | --- |
| Age | | Mean =62.52 | Mean =58.84 | 1.009 (1.003;1.015) | 0.005 |
| Age>65 | | 198/1443 (13.7%) | 1245/1443 (86.3%) | 1.384 (1.120;1.711) | 0.003 |
| Chronic Heart Failure | | 141/661 (21.3%) | 520/661 (78.7%) | 2.249 (1.810;2.794) | <0.001 |
| Coronary artery disease | | 103/620 (16.6%) | 517/620 (83.4%) | 1.471 (1.166;1.856) | 0.001 |
|  |  |  |  |  |  |
| Previous Stroke or TIA | | 55/340 (16.2%) | 285/340 (83.8%) | 1.380 (1.033;1.842) | 0.03 |
|  |  |  |  |  |  |
| Diabetes mellitus | | 111/704 (15.8%) | 593/704 (84.2%) | 1.521 (1.214;1.907) | <0.001 |
|  |  |  |  |  |  |
| Hypertension | | 197/1499 (13.1%) | 1302/1499 (86.9%) | 1.298 (1.052;1.601) | 0.02 |
|  |  |  |  |  |  |
| Congenital heart disease | | 21/365 (5.8%) | 344/365 (94.2%) | 0.479 (0.308;0.745) | 0.001 |
|  |  |  |  |  |  |
| Imaging-PetScan | | 23/518 (4.4%) | 495/518 (95.6%) | 0.302 (0.198;0.462) | <0.001 |
|  |  |  |  |  |  |
| Imaging-MRI | | 38/581 (6.5%) | 543/581 (93.5%) | 0.534 (0.381;0.748) | <0.001 |
|  |  |  |  |  |  |
| Chronic Renal Failure | | 105/551 (19.1%) | 446/551 (80.9%) | 1.820 (1.446;2.290) | <0.001 |
|  |  |  |  |  |  |
| Echo1-LVEF | | Mean =53.04 | Mean =55.96 | 0.986 (0.977;0.994) | <0.001 |
| Location of endocarditis-Aortic | | 186/1514 (12.3%) | 1328/1514 (87.7%) | 1.350 (1.092;1.669) | 0.005 |
|  |  |  |  |  |  |
| Location of endocarditis-ICD/PM | | 30/333 (9.0%) | 303/333 (91.0%) | 0.659 (0.453;0.959) | 0.03 |
|  |  |  |  |  |  |
| Location of endocarditis-Mitral | | 180/1283 (14.0%) | 1103/1283 (86.0%) | 1.532 (1.243;1.890) | <0.001 |
|  |  |  |  |  |  |
| Symptoms - Congestive heart failure | | 167/846 (19.7%) | 679/846 (80.3%) | 2.716 (2.203;3.347) | <0.001 |
|  |  |  |  |  |  |
| Symptoms - Cardiogenic shock | | 16/63 (25.4%) | 47/63 (74.6%) | 3.107 (1.876;5.144) | <0.001 |
|  |  |  |  |  |  |
| Symptoms - Septic shock | | 63/203 (31.0%) | 140/203 (69.0%) | 3.608 (2.743;4.746) | <0.001 |
|  |  |  |  |  |  |
| Symptoms - Janeway lesions | | 18/109 (16.5%) | 91/109 (83.5%) | 1.673 (1.041;2.689) | 0.03 |
|  |  |  |  |  |  |
| Symptoms - Abscess | | 52/363 (14.3%) | 311/363 (85.7%) | 1.463 (1.090;1.965) | 0.01 |
|  |  |  |  |  |  |
| Symptoms - Spondylitis | | 9/168 (5.4%) | 159/168 (94.6%) | 0.422 (0.217;0.818) | 0.01 |
|  |  |  |  |  |  |
| Symptoms - Embolism | | 112/790 (14.2%) | 678/790 (85.8%) | 1.423 (1.138;1.780) | 0.002 |
|  |  |  |  |  |  |
| Symptoms - Cerebral Embolism | | 64/349 (18.3%) | 285/349 (81.7%) | 2.042 (1.556;2.678) | <0.001 |
|  |  |  |  |  |  |
| Symptoms - Hemorrhagic stroke | | 14/67 (20.9%) | 53/67 (79.1%) | 1.862 (1.091;3.178) | 0.02 |
|  |  |  |  |  |  |
| Symptoms - Conduction abnormality on ECG | AV block I | 18/232 (7.8%) | 214/232 (92.2%) | 0.664 (0.413;1.069) | 0.03 |
|  | AV block II | 3/17 (17.6%) | 14/17 (82.4%) | 1.622 (0.520;5.057) |  |
|  | AV block III | 15/82 (18.3%) | 67/82 (81.7%) | 1.782 (1.060;2.994) |  |
| AE - Embolic events | | 123/641 (19.2%) | 518/641 (80.8%) | 2.210 (1.775;2.751) | <0.001 |
|  |  |  |  |  |  |
| AE - CHF | | 106/436 (24.3%) | 330/436 (75.7%) | 3.000 (2.388;3.769) | <0.001 |
|  |  |  |  |  |  |
| AE - Cardiogenic Shock | | 82/189 (43.4%) | 107/189 (56.6%) | 5.516 (4.296;7.081) | <0.001 |
|  |  |  |  |  |  |
| AE - Septic Shock | | 119/287 (41.5%) | 168/287 (58.5%) | 5.920 (4.743;7.388) | <0.001 |
|  |  |  |  |  |  |
| AE - Cerebral haemorrhaege | | 21/71 (29.6%) | 50/71 (70.4%) | 2.908 (1.870;4.520) | <0.001 |
|  |  |  |  |  |  |
| AE - Mycotic aneurysm | | 11/58 (19.0%) | 47/58 (81.0%) | 2.163 (1.184;3.949) | 0.01 |
|  |  |  |  |  |  |
| AE - Acute Renal failure | | 126/548 (23.0%) | 422/548 (77.0%) | 2.787 (2.241;3.465) | <0.001 |
|  |  |  |  |  |  |
| AE - Persistent fever>7days | | 73/350 (20.9%) | 277/350 (79.1%) | 1.947 (1.502;2.523) | <0.001 |
|  |  |  |  |  |  |
| AE - Positive blood cultures after 48h | | 66/412 (16.0%) | 346/412 (84.0%) | 1.668 (1.268;2.195) | <0.001 |
|  |  |  |  |  |  |
| AE - New Abscess | | 32/193 (16.6%) | 161/193 (83.4%) | 1.797 (1.248;2.587) | 0.002 |
|  |  |  |  |  |  |
| AE - AV block | | 19/128 (14.8%) | 109/128 (85.2%) | 1.766 (1.106;2.819) | 0.02 |
|  |  |  |  |  |  |
| AE - Thrombocytopenia<100000/µL | | 53/214 (24.8%) | 161/214 (75.2%) | 2.477 (1.848;3.322) | <0.001 |
|  |  |  |  |  |  |

AE:adverse event; AV:atrioventricular; CI= Confidence Interval; LVEF: left ventricular ejection fraction; MRI: magnetic resonance imaging; PetScan: positron emission tomography scan; ICD/PM: implantable cardioverter defibrillator/pacemaker; TIA= Transient Ischemic Attack. * In order to make the table readable, only the variables significantly associated with the outcome have been reported.

**Supplementary table 5.** Univariable Cox proportional hazards models for mortality at 1-year.

| **Variable** | | **Dead** | **Alive** | **Hazard Ratio(95% CI)** | **P-Value** |
| --- | --- | --- | --- | --- | --- |
| Age | | Mean =63.74 | Mean =57.92 | 1.013 (1.009;1.018) | <0.001 |
| Age>65 | | 415/1443 (28.8%) | 1028/1443 (71.2%) | 1.525 (1.313;1.770) | <0.001 |
|  |  |  |  |  |  |
| BMI | | Mean =26.27 | Mean =25.69 | 1.010 (1.001;1.020) | 0.02 |
| Chronic Heart Failure | | 251/661 (38.0%) | 410/661 (62.0%) | 2.091 (1.787;2.446) | <0.001 |
|  |  |  |  |  |  |
| Coronary artery disease | | 209/620 (33.7%) | 411/620 (66.3%) | 1.540 (1.307;1.814) | <0.001 |
|  |  |  |  |  |  |
| Preexisting valvular disease | | 283/1066 (26.5%) | 783/1066 (73.5%) | 1.171 (1.008;1.361) | 0.04 |
|  |  |  |  |  |  |
| Cancer | | 116/359 (32.3%) | 243/359 (67.7%) | 1.437 (1.177;1.754) | <0.001 |
|  |  |  |  |  |  |
| Previous Stroke or TIA | | 99/340 (29.1%) | 241/340 (70.9%) | 1.243 (1.005;1.539) | 0.04 |
|  |  |  |  |  |  |
| Diabetes mellitus | | 237/704 (33.7%) | 467/704 (66.3%) | 1.682 (1.439;1.967) | <0.001 |
|  |  |  |  |  |  |
| Hypertension | | 410/1499 (27.4%) | 1089/1499 (72.6%) | 1.407 (1.213;1.631) | <0.001 |
|  |  |  |  |  |  |
| Congenital heart disease | | 47/365 (12.9%) | 318/365 (87.1%) | 0.495 (0.368;0.666) | <0.001 |
|  |  |  |  |  |  |
| Previous valve replacement | | 275/1022 (26.9%) | 747/1022 (73.1%) | 1.208 (1.039;1.405) | 0.01 |
|  |  |  |  |  |  |
| Imaging-PetScan | | 84/518 (16.2%) | 434/518 (83.8%) | 0.526 (0.418;0.661) | <0.001 |
|  |  |  |  |  |  |
| Imaging-MRI | | 96/581 (16.5%) | 485/581 (83.5%) | 0.639 (0.515;0.793) | <0.001 |
|  |  |  |  |  |  |
| Chronic Renal Failure | | 220/551 (39.9%) | 331/551 (60.1%) | 2.092 (1.783;2.456) | <0.001 |
|  |  |  |  |  |  |
| Echo1-LVEF | | Mean =53.39 | Mean =56.28 | 0.986 (0.980;0.993) | <0.001 |
| Location of endocarditis-Aortic | | 365/1514 (24.1%) | 1149/1514 (75.9%) | 1.202 (1.036;1.395) | 0.02 |
|  |  |  |  |  |  |
| Location of endocarditis-Mitral | | 337/1283 (26.3%) | 946/1283 (73.7%) | 1.347 (1.163;1.561) | <0.001 |
|  |  |  |  |  |  |
| Location of endocarditis-Unknown | | 7/51 (13.7%) | 44/51 (86.3%) | 0.447 (0.212;0.942) | 0.03 |
|  |  |  |  |  |  |
| Other surgery | | 60/276 (21.7%) | 216/276 (78.3%) | 1.477 (1.097;1.988) | 0.01 |
|  |  |  |  |  |  |
| Symptoms - Congestive heart failure | | 303/846 (35.8%) | 543/846 (64.2%) | 2.439 (2.101;2.830) | <0.001 |
|  |  |  |  |  |  |
| Symptoms - Cardiogenic shock | | 25/63 (39.7%) | 38/63 (60.3%) | 2.526 (1.692;3.773) | <0.001 |
|  |  |  |  |  |  |
| Symptoms - Septic shock | | 96/203 (47.3%) | 107/203 (52.7%) | 2.779 (2.239;3.449) | <0.001 |
| Symptoms - Abscess | | 107/363 (29.5%) | 256/363 (70.5%) | 1.499 (1.220;1.842) | <0.001 |
|  |  |  |  |  |  |
| Symptoms - Spondylitis | | 30/168 (17.9%) | 138/168 (82.1%) | 0.664 (0.460;0.959) | 0.03 |
|  |  |  |  |  |  |
| Symptoms - Embolism | | 200/790 (25.3%) | 590/790 (74.7%) | 1.234 (1.048;1.453) | 0.01 |
|  |  |  |  |  |  |
| Symptoms - Cerebral Embolism | | 108/349 (30.9%) | 241/349 (69.1%) | 1.705 (1.388;2.094) | <0.001 |
|  |  |  |  |  |  |
| Symptoms - Coronary Embolism | | 11/22 (50.0%) | 11/22 (50.0%) | 2.823 (1.555;5.127) | <0.001 |
|  |  |  |  |  |  |
| Symptoms - Hemorrhagic stroke | | 23/67 (34.3%) | 44/67 (65.7%) | 1.657 (1.093;2.510) | 0.02 |
|  |  |  |  |  |  |
| Symptoms - Conduction abnormality on ECG | AV block I | 39/232 (16.8%) | 193/232 (83.2%) | 0.707 (0.511;0.978) | <0.001 |
|  | AV block II | 8/17 (47.1%) | 9/17 (52.9%) | 2.155 (1.073;4.328) |  |
|  | AV block III | 28/82 (34.1%) | 54/82 (65.9%) | 1.811 (1.239;2.647) |  |
| AE - Embolic events | | 197/641 (30.7%) | 444/641 (69.3%) | 1.612 (1.368;1.900) | <0.001 |
|  |  |  |  |  |  |
| AE - CHF | | 182/436 (41.7%) | 254/436 (58.3%) | 2.722 (2.298;3.224) | <0.001 |
|  |  |  |  |  |  |
| AE - Cardiogenic Shock | | 133/189 (70.4%) | 56/189 (29.6%) | 5.785 (4.772;7.013) | <0.001 |
|  |  |  |  |  |  |
| AE - Septic Shock | | 196/287 (68.3%) | 91/287 (31.7%) | 5.857 (4.958;6.919) | <0.001 |
|  |  |  |  |  |  |
| AE - Glomerulonephritis | | 35/89 (39.3%) | 54/89 (60.7%) | 2.130 (1.516;2.993) | <0.001 |
|  |  |  |  |  |  |
| AE - Cerebral haemorrhaege | | 32/71 (45.1%) | 39/71 (54.9%) | 2.328 (1.633;3.319) | <0.001 |
|  |  |  |  |  |  |
| AE - Mycotic aneurysm | | 18/58 (31.0%) | 40/58 (69.0%) | 1.636 (1.024;2.614) | 0.04 |
|  |  |  |  |  |  |
| AE - Acute Renal failure | | 234/548 (42.7%) | 314/548 (57.3%) | 2.725 (2.330;3.187) | <0.001 |
|  |  |  |  |  |  |
| AE - Persistent fever>7days | | 133/350 (38.0%) | 217/350 (62.0%) | 1.859 (1.538;2.248) | <0.001 |
|  |  |  |  |  |  |
| AE - Positive blood cultures after 48h | | 131/412 (31.8%) | 281/412 (68.2%) | 1.571 (1.296;1.906) | <0.001 |
|  |  |  |  |  |  |
| AE - Increasing vegetation size | | 62/201 (30.8%) | 139/201 (69.2%) | 1.522 (1.172;1.977) | 0.002 |
|  |  |  |  |  |  |
| AE - New Abscess | | 68/193 (35.2%) | 125/193 (64.8%) | 1.955 (1.521;2.513) | <0.001 |
|  |  |  |  |  |  |
| AE - Thrombocytopenia<100000/µL | | 103/214 (48.1%) | 111/214 (51.9%) | 2.702 (2.190;3.334) | <0.001 |
|  |  |  |  |  |  |

AE:adverse event; AV:atrioventricular; CHF= Congestive Heart Failure; CI= Confidence Interval; LVEF: left ventricular ejection fraction; MRI: magnetic resonance imaging; PetScan: positron emission tomography scan; TIA= Transient Ischemic Attack. * In order to make the table readable, only the variables significantly associated with the outcome have been reported.

## Supplementary table 6. Indication of surgery on patients with culture positive vs culture negative endocarditis

| **Variable** | | **Total N=3113** | **CPIE N=2590 (83.2%)** | **CNIE N=523 (16.8%)** | **P-Value** |
| --- | --- | --- | --- | --- | --- |
| Theoretical Indication of surgery/procedure | | 2157/3112 (69.3%) | 1797/2590 (69.4%) | 360/522 (69.0%) | 0.85 |
| Surgery performed | | 1596/2157 (74.0%) | 1352/1797 (75.2%) | 244/360 (67.8%) | 0.003 |
| Combination theoretical indication and surgery performed | No theoretical indication | 955/3112 (30.7%) | 793/2590 (30.6%) | 162/522 (31.0%) | 0.01 |
|  | Theoretical indication and surgery performed | 1596/3112 (51.3%) | 1352/2590 (52.2%) | 244/522 (46.7%) |  |
|  | Theoretical indication but surgery not performed | 561/3112 (18.0%) | 445/2590 (17.2%) | 116/522 (22.2%) |  |
| Among patients with theoretical indication: | Surgery performed | 1596/2157 (74.0%) | 1352/1797 (75.2%) | 244/360 (67.8%) | 0.003 |
|  | Surgery not performed | 561/2157 (26.0%) | 445/1797 (24.8%) | 116/360 (32.2%) |  |
| CNIE: culture negative infective endocarditis; CPIE: culture positive infective endocarditis. | | | | | |

**Supplementary table 7.** Sensitivity analyses: multivariable Cox proportional hazard models based on least absolute shrinkage and selection operator (LASSO) models for mortality at 30-days and 1-year (overall and stratified by medical and surgical subgroups).

| **Variable** | **Hazard Ratio*** |
| --- | --- |
| *Mortality at 30 days* | |
| **CNIE** | **1.30** |
| Age | 1.00 |
| Age>65 | 1.07 |
| Congenital disease | 0.87 |
|  |  |
| Ischaemic heart disease (CAD) | 1.06 |
| Previous Stroke / TIA | 1.07 |
| Arterial Hypertension | 1.00 |
|  |  |
| Chronic renal failure | 1.10 |
| Diabetes mellitus | 1.11 |
|  |  |
| Symptoms - CHF | 1.34 |
| Symptoms - Cardiogenic shock | 0.91 |
|  |  |
| Symptoms - Septic shock | 1.19 |
|  |  |
| Symptoms – Abscess | 1.00 |
| Symptoms – Spondylitis | 0.86 |
| Symptoms - Embolic events | 1.00 |
|  |  |
| Symptoms – Cerebral Embolism | 1.59 |
| Symptoms - Hemorrhagic Stroke | 1.00 |
|  |  |
| Symptoms - Conduction abnormality on ECG AV block I  AV block II  AV block III | 0.90  2.08  1.92 |
|  |  |
| Echo1-LVEF | 1.00 |
| AE - Embolic events | 1.59 |
| AE - CHF | 1.55 |
|  |  |
| AE - Cardiogenic shock | 1.73 |
| AE - Septic shock | 2.80 |
|  |  |
| AE - Cerebral haemorrhaege | 2.01 |
| AE - Mycotic aneurysm | 1.00 |
|  |  |
| AE - Acute renal failure | 1.18 |
|  |  |
| AE - Persistent fever | 1.05 |
| AE - Thrombocytopenia<100000/µL | 1.00 |
|  |  |
| Location of Endocarditis – Aortic | 1.39 |
| Location of Endocarditis - ICD/PM | 0.80 |
| Location of Endocarditis - Mitral | 1.35 |
| FDG PET/CT Scan | 0.46 |
| Magnetic Resonance Imaging | 0.53 |
| **Surgery performed** | 0.38 |
|  |  |
| *Mortality at 30 days - Medical therapy subgroup* | |
| **CNIE** | 1.37 |
| Congenital disease | 0.88 |
|  |  |
| Previous Stroke / TIA | 1.08 |
|  |  |
| Chronic renal failure | 1.13 |
|  |  |
| Symptoms - CHF | 1.36 |
|  |  |
| Symptoms - Septic shock | 1.41 |
|  |  |
| Symptoms – Cerebral Embolism | 1.47 |
|  |  |
| Symptoms - Conduction abnormality on ECG AV block III | 2.23 |
|  |  |
| Echo1-LVEF | 1.00 |
|  |  |
| AE - Embolic events | 1.37 |
|  |  |
| AE - CHF | 1.76 |
| AE - Cardiogenic shock | 1.48 |
|  |  |
| AE - Septic shock | 2.90 |
|  |  |
| AE - Cerebral haemorrhaege | 1.64 |
|  |  |
| AE - Acute renal failure | 1.14 |
|  |  |
| AE - Persistent fever | 1.03 |
| AE - New Abscess | 0.91 |
| AE - AV block | 1.18 |
| Location of Endocarditis – Aortic | 1.50 |
| Location of Endocarditis - ICD/PM | 0.90 |
| Location of Endocarditis - Mitral | 1.43 |
| FDG PET/CT Scan | 0.35 |
| Magnetic Resonance Imaging | 0.60 |
| *Mortality at 30 days - Surgery subgroup* | |
| **CNIE** | 1.23 |
| Age>65 | 1.27 |
| Janeway lesion | 0.69 |
| Ischaemic heart disease (CAD) | 1.30 |
| Arterial Hypertension | 1.11 |
| Chronic renal failure | 1.05 |
| Diabetes mellitus | 1.70 |
| Symptoms - CHF | 1.15 |
| Symptoms - Cardiogenic shock | 0.67 |
| Symptoms - Septic shock | 0.62 |
| Symptoms – Abscess | 1.07 |
| Symptoms – Spondylitis | 0.83 |
| Symptoms - Embolic events | 1.15 |
| Symptoms – Cerebral Embolism | 1.35 |
| Symptoms - Conduction abnormality on ECG AV block I  AV block II | 0.77  5.19 |
| Echo1-LVEF | 1.01 |
| AE - Embolic events | 1.66 |
| AE - Cardiogenic shock | 2.93 |
| AE - Septic shock | 2.21 |
| AE - Cerebral haemorrhaege | 4.82 |
| AE - Acute renal failure | 1.24 |
| AE – Positive blood cultures 48h after surgery | 0.92 |
| AE – New abscess | 1.50 |
| Location of Endocarditis - ICD/PM | 0.95 |
| Magnetic Resonance Imaging | 0.50 |
| *Mortality at 1 year* | |
| **CNIE** | **1.24** |
| Age | 1.00 |
| Congenital disease | 0.79 |
|  |  |
| Previous valvular intervention | 1.12 |
| Previous Stroke / TIA | 1.06 |
| Chronic renal failure | 1.20 |
| Diabetes mellitus | 1.26 |
|  |  |
| History of cancer | 1.09 |
| Symptoms - CHF | 1.32 |
| Symptoms - Cardiogenic shock | 0.87 |
|  |  |
| Symptoms – Abscess | 0.99 |
| Symptoms – Spondylitis | 0.83 |
| Symptoms – Cerebral Embolism | 1.49 |
| Symptoms – Coronary embolism | 2.09 |
|  |  |
| Symptoms - Conduction abnormality on ECG AV block I  AV block II  AV block III | 0.77  2.79  1.79 |
|  |  |
| AE - Embolic events | 1.09 |
| AE - CHF | 1.54 |
|  |  |
| AE - Cardiogenic shock | 1.67 |
| AE - Septic shock | 3.36 |
|  |  |
| AE - Cerebral haemorrhaege | 2.11 |
| AE - Glomerulonephritis | 0.96 |
|  |  |
| AE - Acute renal failure | 1.39 |
|  |  |
| AE - Persistent fever | 1.22 |
| AE - Thrombocytopenia<100000/µL | 1.39 |
|  |  |
| AE – Increasing vegetation size | 1.15 |
| AE – New abscess | 1.13 |
| Location of Endocarditis – Aortic | 1.18 |
| Location of Endocarditis - Mitral | 1.14 |
| FDG PET/CT Scan | 0.76 |
| Magnetic Resonance Imaging | 0.75 |
| **Surgery performed** | 0.44 |
|  |  |
| *Mortality at 1 year - Medical therapy subgroup* | |
| **CNIE** | 1.20 |
| Age | 1.00 |
| Congenital disease | 0.95 |
|  |  |
| Previous Stroke / TIA | 1.12 |
|  |  |
| Chronic renal failure | 1.07 |
|  |  |
| Diabetes | 1.12 |
| History of cancer | 1.22 |
| Symptoms - CHF | 1.42 |
|  |  |
| Symptoms - Septic shock | 1.09 |
|  |  |
| Symptoms - Abscess | 0.78 |
| Symptoms – Spondylitis | 0.89 |
| Symptoms – Cerebral Embolism | 1.51 |
|  |  |
| Symptoms – Coronary Embolism | 1.54 |
| Symptoms - Conduction abnormality on ECG AV block I  AV block II  AV block III | 0.92  2.31  2.36 |
|  |  |
| AE - Embolic events | 1.02 |
|  |  |
| AE - CHF | 1.74 |
| AE - Cardiogenic shock | 1.76 |
|  |  |
| AE - Septic shock | 3.66 |
|  |  |
| AE - Cerebral haemorrhaege | 1.99 |
|  |  |
| AE - Acute renal failure | 1.31 |
|  |  |
| AE - Persistent fever | 1.25 |
| AE – Increasing vegetation size | 1.27 |
| AE - Thrombocytopenia<100000/µL | 1.35 |
| Location of Endocarditis – Aortic | 1.32 |
| Location of Endocarditis - Mitral | 1.21 |
| FDG PET/CT Scan | 0.58 |
| Magnetic Resonance Imaging | 0.81 |
| *Mortality at 1 year - Surgery subgroup* | |
| **CNIE** | **1.20** |
| Age>65 | 1.12 |
| Congenital disease | 0.70 |
|  |  |
| Pre-existing valve disease | 1.31 |
| Arterial Hypertension | 1.08 |
|  |  |
| Chronic renal failure | 1.64 |
| Diabetes mellitus | 1.42 |
|  |  |
| Body mass index (Kg/m^2^) | 1.01 |
| Symptoms - CHF | 1.09 |
| Symptoms - Cardiogenic shock | 0.66 |
|  |  |
| Symptoms - Septic shock | 0.95 |
|  |  |
| Symptoms – Abscess | 1.04 |
| Symptoms – Cerebral Embolism | 1.10 |
| Symptoms – Coronary Embolism | 2.38 |
| Symptoms - Hemorrhagic Stroke | 1.12 |
|  |  |
| Symptoms - Conduction abnormality on ECG AV block I  AV block II  AV block III | 0.70  1.75  1.15 |
|  |  |
| AE - Embolic events | 1.07 |
| AE - Cardiogenic shock | 1.59 |
| AE - Septic shock | 2.53 |
|  |  |
| AE - Cerebral haemorrhaege | 2.31 |
| AE - Acute renal failure | 1.37 |
|  |  |
| AE – New abscess | 1.95 |
| AE - Persistent fever | 1.09 |
| AE - Thrombocytopenia<100000/µL | 1.24 |
|  |  |
| FDG PET/CT Scan | 1.01 |
| Magnetic Resonance Imaging | 0.74 |
| AE:adverse event; AV:atrioventricular; CHF= Congestive Heart Failure; CNIE: culture negative infective endocarditis; LVEF: left ventricular ejection fraction; PetScan: positron emission tomography scan; TIA= Transient Ischemic Attack. * In order to make the table readable, only the variables significantly associated with the outcome have been reported; 95% confidence intervals and p values were not reported as the assumptions of the post-selection inference method were not respected. | |

**Supplementary table 8.** Sensitivity analysis: comparison between Staphylococcus aureus and culture negative endocarditis.

| **Variable** | **Total**  **N=3113** | **Staphilococcus aureus IE**  **N=763 (59.3%)** | **CNIE**  **N=523 (40.7%)** | **P-Value** |
| --- | --- | --- | --- | --- |
| Embolic events | 281/1286 (21.9%) | 201/763 (26.3%) | 80/523 (15.3%) | <0.001 |
| Spondylitis | 52/1286 (4.0%) | 45/763 (5.9%) | 7/523 (1.3%) | <0.001 |
| Cardiogenic shock | 93/1183 (7.9%) | 53/683 (7.8%) | 40/500 (8.0%) | 0.88 |
| Septic shock | 159/1286 (12.4%) | 118/763 (15.5%) | 41/523 (7.8%) | <0.001 |
| Glomerulonephritis | 49/1279 (3.8%) | 29/756 (3.8%) | 20/523 (3.8%) | 0.99 |
| Cerebral haemorrhage | 42/1286 (3.3%) | 32/763 (4.2%) | 10/523 (1.9%) | 0.02 |
| Mycotic aneurysm | 21/1286 (1.6%) | 11/763 (1.4%) | 10/523 (1.9%) | 0.51 |
| Acute renal failure | 255/1286 (19.8%) | 148/763 (19.4%) | 107/523 (20.5%) | 0.61 |
| Persistent fever | 183/1183 (15.5%) | 120/683 (17.6%) | 63/500 (12.6%) | 0.02 |
| Increasing vegetation size | 97/1286 (7.5%) | 66/763 (8.7%) | 31/523 (5.9%) | 0.07 |
| New abscess | 72/1286 (5.6%) | 53/763 (6.9%) | 19/523 (3.6%) | 0.01 |
| AV block | 52/1183 (4.4%) | 35/683 (5.1%) | 17/500 (3.4%) | 0.15 |
| Thrombocytopenia<100000/µL | 100/1183 (8.5%) | 67/683 (9.8%) | 33/500 (6.6%) | 0.05 |
| Heart failure | 159/1013 (15.7%) | 84/595 (14.1%) | 75/418 (17.9%) | 0.10 |
| Valve or prosthetic dysfunction | 175/1014 (17.3%) | 88/596 (14.8%) | 87/418 (20.8%) | 0.01 |
| Death at 30 days | 183/1286 (14.2%) | 105/763 (13.8%) | 78/523 (14.9%) | 0.56 |
| In-hospital death | 272/1286 (21.2%) | 167/763 (21.9%) | 105/523 (20.1%) | 0.44 |
| Death at 1 year | | | | P at 1 year* |
| Overall | 355/1286 (27.6%) | 220/763 (28.8%) | 135/523 (25.8%) | 0.23 |
| Medical therapy | 249/752 (33.1%) | 155/466 (33.3%) | 94/286 (32.9%) | 0.91 |
| Surgical therapy | 101/524 (19.3%) | 64/295 (21.7%) | 37/229 (16.2%) | 0.11 |

Abbreviations: AV: atrioventricular; CNIE: culture negative infective endocarditis; IE : infective endocarditis.

*Results from actuarial survival curves and log-rank tests.
